# Crystal structure of a photosynthetic LH1-RC in complex with its electron donor HiPIP

**DOI:** 10.1038/s41467-021-21397-9

**Published:** 2021-02-17

**Authors:** Tomoaki Kawakami, Long-Jiang Yu, Tai Liang, Koudai Okazaki, Michael T. Madigan, Yukihiro Kimura, Zheng-Yu Wang-Otomo

**Affiliations:** 1 Faculty of Science, Ibaraki University, Mito, Japan; 2 Photosynthesis Research Center, Key Laboratory of Photobiology, Institute of Botany, Chinese Academy of Sciences, Beijing, China; 3 Department of Microbiology, Southern Illinois University, Carbondale, IL USA; 4 Department of Agrobioscience, Graduate School of Agriculture, Kobe University, Nada Kobe, Japan

**Keywords:** Biophysical chemistry, Bioenergetics, X-ray crystallography

## Abstract

Photosynthetic electron transfers occur through multiple components ranging from small soluble proteins to large integral membrane protein complexes. Co-crystallization of a bacterial photosynthetic electron transfer complex that employs weak hydrophobic interactions was achieved by using high-molar-ratio mixtures of a soluble donor protein (high-potential iron-sulfur protein, HiPIP) with a membrane-embedded acceptor protein (reaction center, RC) at acidic pH. The structure of the co-complex offers a snapshot of a transient bioenergetic event and revealed a molecular basis for thermodynamically unfavorable interprotein electron tunneling. HiPIP binds to the surface of the tetraheme cytochrome subunit in the light-harvesting (LH1) complex-associated RC in close proximity to the low-potential heme-1 group. The binding interface between the two proteins is primarily formed by uncharged residues and is characterized by hydrophobic features. This co-crystal structure provides a model for the detailed study of long-range trans-protein electron tunneling pathways in biological systems.

## Abstract (web summary)

The high potential iron-sulfur (HiPIP) proteins are direct electron donors to the light-harvesting-reaction center complexes (LH1-RC) in photosynthetic β- and γ-*Proteobacteria*. Here, the authors present the 2.9 Å crystal structure of the HiPIP-bound LH1-RC complex from the thermophilic purple sulfur bacterium *Thermochromatium tepidum* and discuss mechanistic implications for the electron transfer pathway.

## Introduction

Photosynthetic organisms oxidize either water or reduced organic or inorganic compounds as electron sources to produce new biomass. While noncyclic electron transport is the major pathway in oxygen-evolving photosynthetic organisms, electron transport in anoxygenic phototrophic bacteria is characterized by a light-driven cyclical process coupled to proton translocation^1^. In purple phototrophic bacteria, the cyclic electron transport chain is composed of three major components, two membrane-embedded protein complexes—reaction center (RC) and cytochrome (Cyt) *bc*_1_—and a group of soluble proteins that shuttle electrons between the RC and Cyt *bc*_1_ complexes. In most purple bacteria, the RC contains a tightly bound multi-heme Cyt subunit that functions as the immediate electron donor to the special pair bacteriochlorophylls (BChl) and as an acceptor from the soluble carriers^2,3^. A variety of soluble *c*-type cytochromes and high-potential iron-sulfur proteins (HiPIP) function as electron donors to the RC^4,5^. For many purple bacteria of the α-proteobacterial group^6^, Cyt *c*_2_ is the sole or major electron donor. This is the case for *Rhodobacter* (*Rb*.) *sphaeroides*, for example, where Cyt *c*_2_ transfers electrons from Cyt *bc*_1_ to the RC. The *Rb. sphaeroides* Cyt *c*_2_-bound RC complex has been crystallized and its structure determined^7,8^. The structure showed that Cyt *c*_2_ is positioned at the center of the periplasmic surface of the RC and that the binding site contains both long-range electrostatic interactions to facilitate rapid association and short-range hydrophobic interactions to facilitate electron tunneling.

By contrast to electron flow in photosynthetic α-*Proteobacteria*, the HiPIP functions as a direct electron donor to the RC in photosynthetic β- and γ-*Proteobacteria*^9,10^. Until now, no structure has been determined for an electron transfer complex formed between a soluble carrier and the RC bound with a multi-heme Cyt subunit. We present here the crystallographic structure of an HiPIP-bound RC in which the RC contains the tetraheme Cyt subunit and is associated with its core light-harvesting complex (LH1-RC, ~390 kDa). Both the HiPIP and LH1-RC were isolated from the thermophilic purple sulfur bacterium *Thermochromatium* (*Tch*.) *tepidum* (γ-*Proteobacteria*)^11^ and their individual structures have been determined to high resolutions^12,13^. Our structural results verify predictions of protein–protein interactions between the *Tch. tepidum* tetraheme Cyt subunit and HiPIP and go well beyond this to provide important new insight into the mechanism of electron transfer that occurs in this key step of photosynthetic energy generation.

## Results

Both a high molar ratio of *Tch. tepidum* HiPIP to LH1-RC and acidic pH were required for co-crystal formation based on MALDI/TOF-MS measurements taken at the initial stage of screening (Supplementary Figs. 1 and 2). Further screenings of the two parameters by analyzing diffraction data over the ranges of HiPIP:LH1-RC = 1–30 and pH 4.5–7.5 revealed the optimum conditions to be near a ratio of 15 and at pH 5.0 (data not shown) as described in the “Methods”. Based on the absorption spectrum (Supplementary Fig. 1), *Tch. tepidum* HiPIP was purified in the reduced state.

### Structural overview

**Fig. 1:**
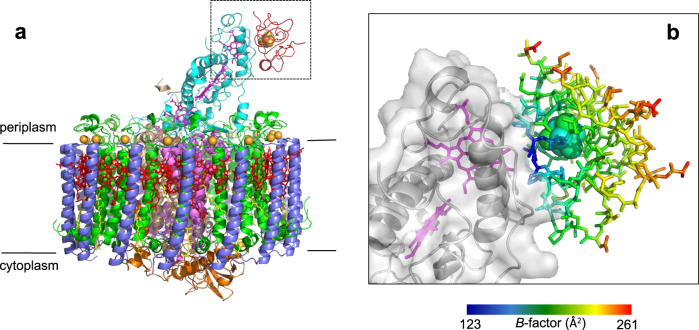
Structure of the HiPIP:LH1-RC co-complex. **a** Side view of the overall structure of the HiPIP:LH1-RC co-complex. Color scheme: HiPIP, red; LH1-α, green; LH1-β, slate blue; RC-Cyt subunit, cyan; RC-L subunit, violet; RC-M subunit, white tint; RC-H subunit, orange; BChl *a*, red sticks; Spirilloxanthin, yellow sticks; heme, magenta sticks; Fe-S, yellow-orange ball; Ca, gold ball. **b** Expanded view of the region marked in **a** where HiPIP is shown as sticks and Cyt subunit is shown in transparent surface representation. Hemes are shown in magenta sticks and 4Fe-4S cluster is shown as balls. The residues in HiPIP are color-coded by the values of their *B*-factor as indicated in the scale bar.

The crystal structure of the HiPIP:LH1-RC co-complex was determined at 2.9 Å resolution. Each LH1-RC bound one HiPIP at the surface of the Cyt subunit, covering a range from Glu60 to Asp120 and in close proximity to the low-potential heme-1 (Fig. 1 and Supplementary Fig. 3). The amino acid sequences of these proteins in *Tch. tepidum* were compared with those of other species in Supplementary Fig. 3. The contact surface area between the two proteins was calculated to be 667 Å^2^, similar in order of magnitude to that of the Cyt *c*_2_:RC co-complex of the purple bacterium *Rb. sphaeroides*^8^. Upon binding to the Cyt subunit, the HiPIP exhibited an uneven *B*-factor distribution with smaller values for the amino acid residues on the binding interface where Gln62 and Leu63 showed the smallest *B*-factors (Fig. 1b and Supplementary Fig. 4). This indicates that the interface residues of HiPIP have significantly more restricted molecular motions compared with those on the opposite side exposed to the solvent.

**Fig. 2:**
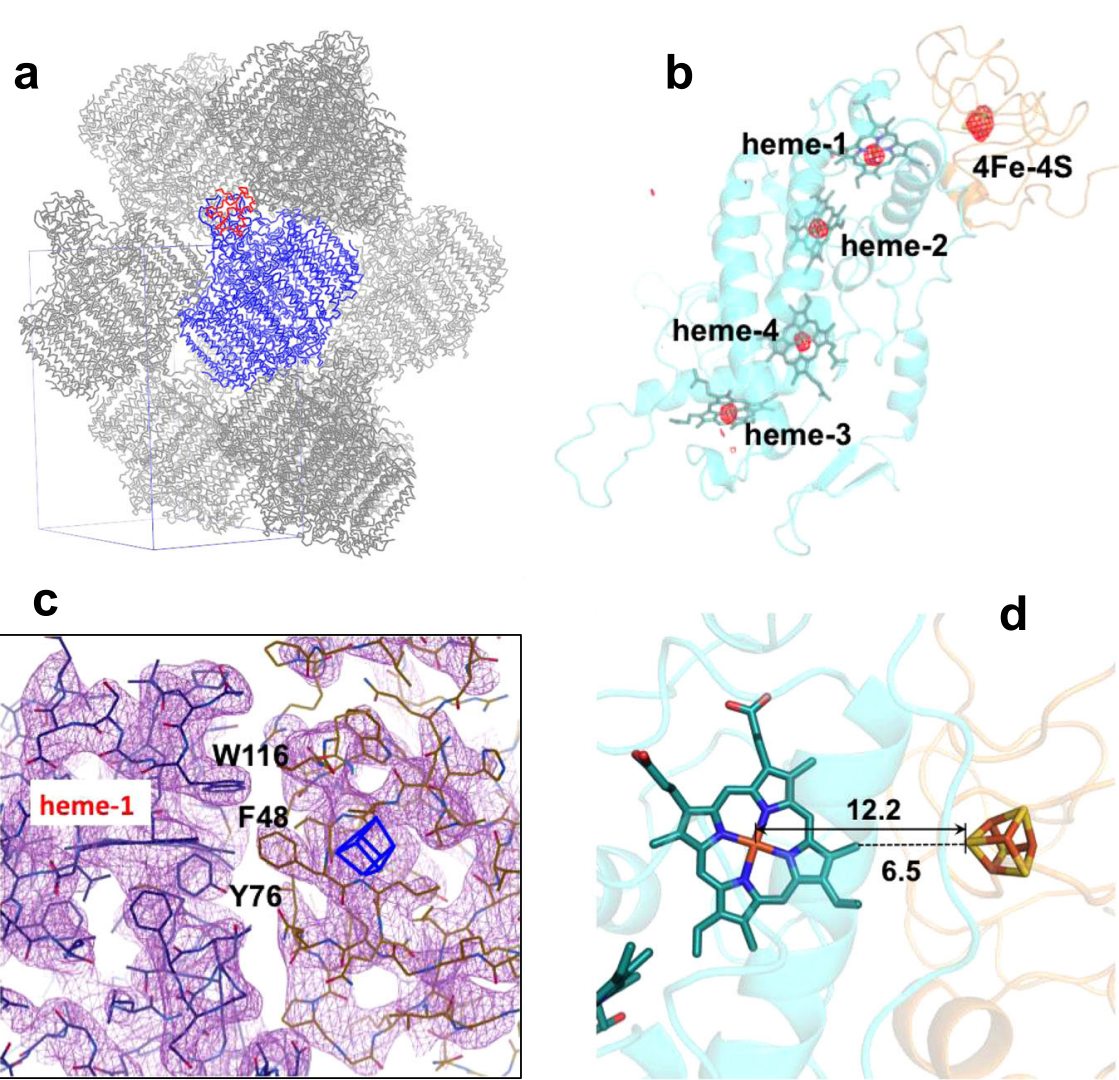
Structural arrangements of the HiPIP:LH1-RC co-complex at different scales. **a** Crystal packing of the LH1-RC (blue) and HiPIP (red). The rectangular box (blue lines) shows a unit cell and each unit cell contains one HiPIP:LH1-RC. **b** Anomalous difference Fourier maps measured at 0.9 Å for the C-subunit and HiPIP. Red meshes represent the contour levels around Fe atoms at 4.2*σ*. **c** Electron density maps at a contour level of 1.4*σ* for the interface area between HiPIP (right) and Cyt subunit (left). The 4Fe-4S cluster is shown by a blue box. This figure was drawn using Coot software. **d** Distances (Å) between the 4Fe-4S cluster in HiPIP and the heme-1 in the Cyt subunit.

The *Tch. tepidum* HiPIP:LH1-RC co-complex had a more compact crystal packing than did the LH1-RC alone, as indicated by reductions of both the solvent content from 61.9 to 57.1% and the Matthew coefficient from 3.22 to 2.97 Å^3^/Da (Fig. 2a). The positions of the Fe atoms in both hemes and the 4Fe-4S cluster were clearly resolved from the strong anomalous difference Fourier map (Fig. 2b). Most sidechains of HiPIP residues could be modeled based on the electron density map with higher accuracies for the interface residues because of their lower *B*-factors (Fig. 2c). The interface between the two proteins displayed a zigzag shape in which the large sidechains of Tyr76 and Trp116 of the Cyt subunit and Phe48 of HiPIP likely function as shape-recognition residues. The nearest distance between the two prosthetic groups was 6.5 Å as measured from the sulfur S1 in HiPIP to the 2^1^-methyl carbon of heme-1, and the distance between S1 and Fe (heme-1) was 12.2 Å (Fig. 2d). Coordinate error was estimated to be 0.50 Å based on the maximum-likelihood refinement^14^.

### Interactions between *Tch. tepidum* HiPIP and Cyt subunit

**Fig. 3:**
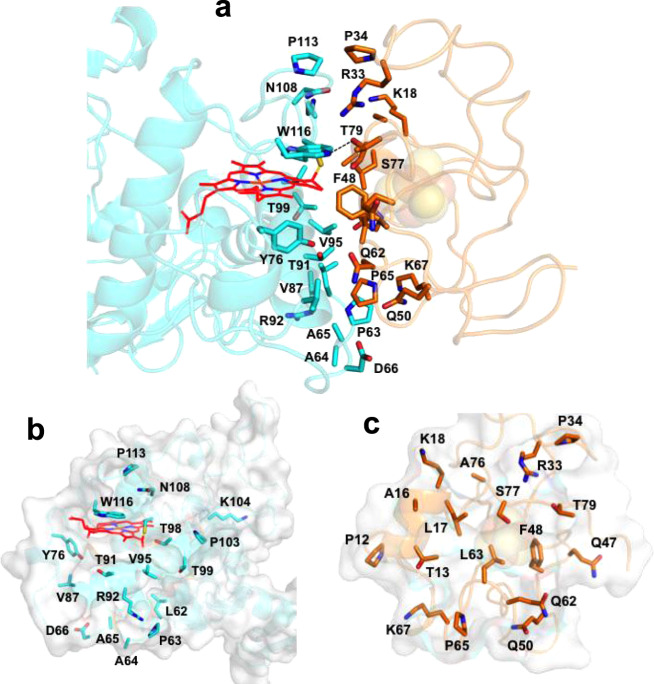
Amino acid residues on the interface of HiPIP (orange) and the Cyt subunit (cyan). Side view (**a**) and front views of the Cyt subunit (**b**) and HiPIP (**c**). A hydrogen bond formed between Trp116 (Cyt subunit) and Thr79 (HiPIP) is shown by a dashed line in **a**. Sidechains of the residues are shown in sticks. Protein structures are drawn by transparent cartoons. Macrocycle of the heme-1 in the Cyt subunit is shown as red framework. Color scheme: oxygen, red; nitrogen, blue; sulfur, yellow.

Figure 3 shows the amino acid residues on the interface of the *Tch. tepidum* HiPIP and Cyt subunit. Most of the residues are distributed around the 4Fe-4S cluster and heme-1. The path between the two prosthetic groups is mainly composed of uncharged residues: Thr13, Leu17, Phe48, Leu63, and Ser77 in the HiPIP (Fig. 3c) and Tyr76, Thr91, Val95, Thr98, and Trp116 in the Cyt subunit (Fig. 3b). The closest distance between the two proteins was 2.3 Å as measured for the hydroxyl oxygen of Thr79 (HiPIP) and the aromatic nitrogen of Trp116 (Cyt subunit) which form a strong hydrogen bond (Fig. 3a and Supplementary Table 2). Other relatively weak hydrogen bonds may be formed between Arg33(HiPIP)/Asn108(Cyt subunit), Gln62(HiPIP)/Tyr76(Cyt subunit), and Gln62(HiPIP)/Thr91(Cyt subunit) (Supplementary Table 2).

It is notable that the binding surface of HiPIP is characterized by its hydrophobicity as indicated by the electrostatically neutral distribution formed by uncharged residues (Supplementary Fig. 5). As a result, the surface charge distribution is virtually unaffected by a change of pH (Supplementary Fig. 5a, b). In contrast, the opposite side of the binding surface of HiPIP is largely acidic at pH 7 (Supplementary Fig. 5c) and apparently shifts toward neutral at pH 5 (Supplementary Fig. 5d). By contrast, the binding surface of the Cyt subunit is relatively basic at pH 7 (Supplementary Fig. 5e), especially near heme-1, and becomes even more basic at pH 5 (Supplementary Fig. 5f). Considering that the HiPIP:LH1-RC co-crystal was preferentially formed at pH 5 and at high molar ratios of HiPIP to LH1-RC, the results imply that interactions between the acidic side of HiPIP (Supplementary Fig. 5c) and the basic surface of the Cyt subunit may weaken at acidic pH, thus increasing the probability of binding of the two proteins at the correct position required for efficient electron transfer.

### Structural changes upon binding of *Tch. tepidum* HiPIP to LH1-RC

**Fig. 4:**
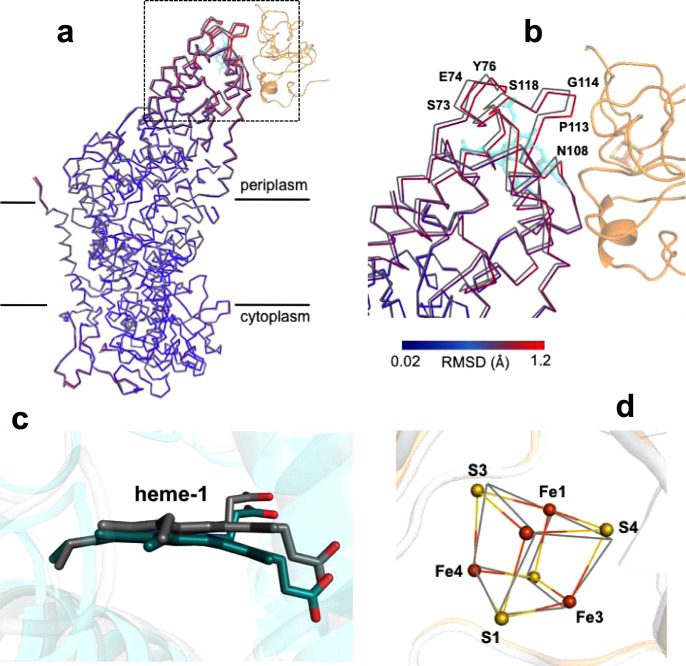
Structural changes in the HiPIP:LH1-RC co-complex. **a** Partial structures for the RC portions obtained by superposition of the Cα carbons of the reaction center L-subunit in HiPIP:LH1-RC (colored) with those in free LH1-RC (gray, PDB ID: 5Y5S). Part of the HiPIP is shown in transparent orange cartoon, and the heme-1 in Cyt subunit is shown in transparent cyan sticks. **b** Expanded view of the region marked in **a** with several residues indicated that have deviations larger than 1.0 Å. The Cα traces of RC are color-coded by the values of root-mean-square deviation (RMSD) as indicated in the scale bar. **c** Side view of superposition of the carbon atoms of the pyrrole ring I in heme-1 (deepteal color) with those in free LH1-RC (gray, PDB ID: 5Y5S). **d** Structural changes of the 4Fe-4S cluster in the HiPIP:LH1-RC co-complex obtained by superposition of the Cα carbons of the HiPIP (colored) with those in free HiPIP (gray, PDB ID: 1EYT). Sulfur and Fe atoms are shown by yellow and orange balls, respectively.

Superposition of the Cα carbons of the membrane-embedded RC L-subunit in the co-complex with those in free LH1-RC revealed that conformational changes mainly occurred on the binding surface of the Cyt subunit, with a maximum deviation of 1.2 Å (Fig. 4a, b). Residues closer to the surface displayed larger deviations from those of free LH1-RC, and large changes in position were observed for the residues of Thr68–Asn78 and Asn108–Ser118 around the heme-1-binding site in the Cyt subunit (Supplementary Fig. 3). No detectable conformational changes were found in the LH1 complex.

A remarkable change was observed for heme-1 in the Cyt subunit of the co-complex (Fig. 4c). The heme-1 macrocycle displayed a significantly bent conformation along the diagonal of pyrrole rings I and III compared to that of heme-1 in the free LH1-RC (Supplementary Fig. 6a). Superposition of the pyrrole rings I of the heme-1 groups revealed a maximum deviation of 1.3 Å at the edge of pyrrole III (Fig. 4c). This feature may be related to the function of heme-1 because pyrrole ring I is nearest the 4Fe-4S cluster in HiPIP (Fig. 2d) and is predicted as the entry point for electrons donated by the 4Fe-4S cluster. By contrast, essentially no change was found for the conformation of HiPIP (Supplementary Fig. 6b). When superimposing the Cα carbons of the HiPIP in co-complex with those in free HiPIP, only a slight rotation was observed for the 4Fe-4S cluster (Fig. 4d), with a maximum deviation of 0.4 Å at sulfur S4. Moreover, no relative changes in distances and angles among the atoms in the cluster were found (Supplementary Fig. 6c).

### Predicted electron transfer pathway

**Fig. 5:**
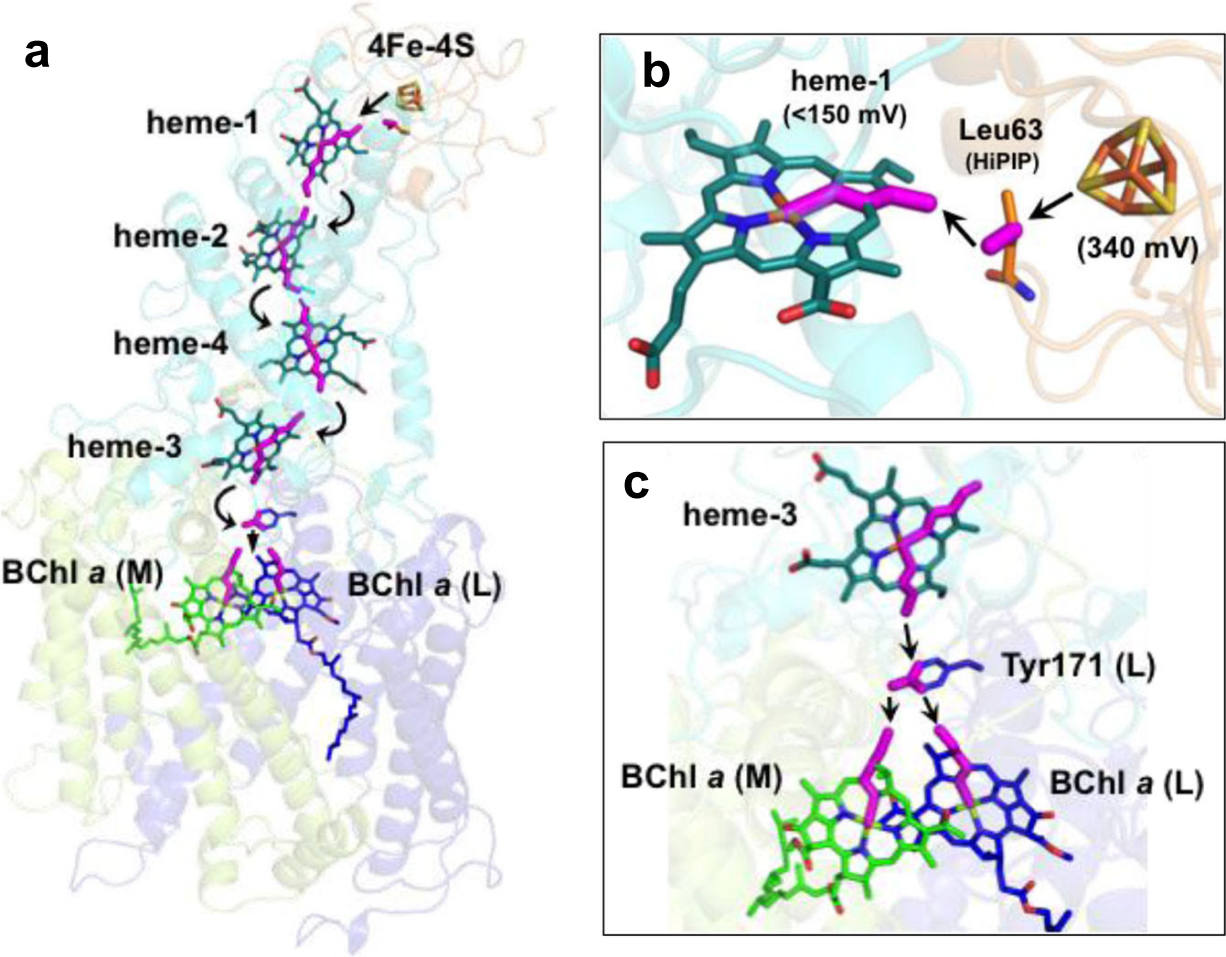
Predicted electron transfer pathways. **a** Trans-protein electron tunneling pathway from 4Fe-4S cluster to the special pair predicted by the *Pathways* plugin for VMD using standard parameters. Through-bond process and through-space hoppings are represented by thick magenta sticks and black arrows, respectively. **b** Expanded view of the portion of 4Fe-4S cluster in HiPIP and heme-1 in the Cyt subunit. Leu63 in HiPIP is shown by sticks. Reduction potentials are given in parentheses. **c** Expanded view of the portion of heme-3 in the Cyt subunit and the special pair BChl *a* dimer. The sidechain of the Tyr171 in the reaction center L-subunit is shown by sticks.

From the co-crystal structure, we could construct a five-step trans-protein electron tunneling chain from the HiPIP to the special pair of the RC via the tetraheme Cyt subunit—a distance that spans more than 70 Å (Fig. 5a and Supplementary Fig. 7). The center-to-center distances between each redox pair are in the range of 12.2–20.6 Å as given in Supplementary Table 3. Based on *Pathways* calculations^15^, the first step from 4Fe-4S to heme-1 is mediated by the sidechain of Leu63 in HiPIP, which is located in the middle, 3.5 Å away from both sulfur S1 in HiPIP and 2^1^-methyl carbon in heme-1 (Fig. 5b). Based on its maximum coupling strength and close proximity to the Fe in heme-1, sulfur S1 was predicted as the atom in the 4Fe-4S cluster that donates electrons to heme-1 (Supplementary Table 3). The putative pathway within the Cyt subunit is an inter-heme electron tunneling process (center-to-center distances of 13.9–16.2 Å) with relatively greater coupling strengths compared to that of S1-to-heme-1 (Supplementary Table 3). The electrons in heme-1 were transferred along pyrrole rings I and II to the heme-2, with an edge-to-edge distance of 4.0 Å. While pyrrole rings I and II also participate in the electron paths in heme-2 and heme-3, the route in heme-4 was predicted as passing along the pyrrole rings II and IV. It is unclear whether the different routes are related to different coordinations of the central Fe atoms in which the Fe atoms in heme-1, heme-2, and heme-3 are ligated by His and Met residues whereas the Fe atom in heme-4 is ligated by two His residues. The last step of electron transfer to the special pair is relayed by the aromatic ring of Tyr171 in the L-subunit, which is located directly above the BChl *a* dimer (Fig. 5c). This Tyr residue is conserved in both Cyt subunit-possessing (*Blastochloris viridis* and *Rubrivivax gelatinosus*) and Cyt subunit-deficient (*Rb. sphaeroides*) species of purple bacteria. In the Cyt *c*_2_:RC co-crystal structure of *Rb. sphaeroides*, the corresponding Tyr(L162) was identified as the bridging residue for electron transfer from the heme group in Cyt *c*_2_ to the special pair^8^, and the heme plane is largely overlapped with that of heme-3 in the *Tch. tepidum* Cyt subunit. The final pathways to the central Mg atoms of the BChl *a* dimer in the L- and M-subunits were predicted to pass through pyrrole ring II in both BChl *a* molecules.

## Discussion

Approximately equimolar mixtures of proteins have been used in co-crystallizations that mainly employ electrostatic interactions^8,16–20^. In contrast, the high molar ratios (≥10) of HiPIP to LH1-RC were required for successful co-crystallization in our work. The requirement could be due to the weak interactions that allow for rapid equilibrium between the two proteins and exclude formation of a long-lived complex^21^. The high ratios needed likely increase the chance of forming an encounter complex because the HiPIP-to-RC electron transfer is a collisional reaction governed by hydrophobic forces^21,22^. Additionally, an acidic pH was also required for the formation of co-crystals; these conditions probably masked the negative charges on the protein surfaces (Supplementary Fig. 5), thus enhancing hydrophobic interactions. The hydrophobic nature of the interactions is evidenced by both the uncharged feature of the HiPIP-binding surface (Supplementary Fig. 5) and the ionic strength-dependence of electron transfer measurements^21^. This was confirmed by isothermal titration calorimetry (ITC) analysis that revealed positive peaks (endothermic heat change) for the binding of HiPIP to LH1-RC (Supplementary Fig. 8), indicating an entropically driven process. The entropy-driven signature is typically attributed to hydrophobic interactions accompanied by the release of bound water molecules or ions along with conformational changes^23,24^. Such has also been reported for the binding of ferredoxin, a water-soluble protein containing an iron-sulfur cluster, to photosystem I^25^ and ferredoxin-NADP^+^-reductase^26,27^ in oxygenic phototrophs.

The ultra-high-resolution structure (0.48 Å) of *Tch. tepidum* HiPIP revealed details for the charge distribution around its 4Fe-4S cluster at levels of iron 3*d* and sulfur 3*p* orbital electrons^12^. Charge-density analysis yielded a total atomic charge of –1.5 for the 4Fe-4S–4(Cys-Sγ) group, a value close to the total charge of this cluster in the reduced state. Based on an individual valence-charge analysis, the Fe1 (with its ligand Cys43-Sγ), Fe2, and S4 atoms were identified to be crucial for storing electron charges in the reduced free HiPIP^12^. In the *Tch. tepidum* HiPIP:LH1-RC structure, most of these atoms in HiPIP are located on the distal side of the binding surface (Fig. 4d), indicating a role as sources of electrons to the front-side (solvent-exposed) Fe and S atoms that have been proposed to form a delocalized 2Fe–2S subcluster in HiPIP^28^.

The reduction potential of the *Tch. tepidum* HiPIP was determined to be +340 mV^29^, and the two low-potential hemes (heme-1 and heme-4) in the *Tch. tepidum* Cyt subunit were estimated to have reduction potentials below +150 mV^30^. This points to a thermodynamically unfavorable electron transfer from HiPIP to heme-1, followed by a down–up–down–down flow to the special pair in the RC (Fig. 5a)^31,32^. This “uphill” nature of HiPIP oxidation has fueled debate over which heme group in the tetraheme Cyt subunit is actually reduced by HiPIP; for example, heme-2 is more electropositive (~+330 mV) than heme-1 and would therefore be a more thermodynamically favorable acceptor of electrons from HiPIP than would heme-1. However, despite its lower reduction potential, heme-1 has been convincingly shown to be the direct electron acceptor from HiPIP^21,22,33^ and various *c*-type cytochromes^34–36^. Using an empirical formula for the endergonic tunneling proposed using fixed geometry by Page et al.^37^, the rate of electron transfer from the 4Fe-4S cluster of *Tch. tepidum* HiPIP to heme-1 was calculated to be 1.5 × 10^5^ s^−1^ assuming a reorganization energy of 0.7 eV^37^, a volume fraction of 0.77, and a reduction potential of +10 mV for heme-1 derived from *Allochromatium* (*Alc*.) *vinosum*^38^, a mesophilic relative of *Tch. tepidum*. Despite the endergonic nature of this first step, the calculated rate is about four orders of magnitude faster than the measured value (~20 s^−^^1^) for the *Tch. tepidum* HiPIP/RC system in the range of micromolar concentrations^29^. A similar pseudo-first-order rate constant was also observed from the *Alc. vinosum* HiPIP/RC system^21^. These results indicate that complex formation of HiPIP and LH1-RC is the rate-limiting step for the interprotein electron transfer and that the electron transfer from 4Fe-4S to heme-1 is the rate-limiting step in the entire pathway to the special pair in the RC.

A significant distortion of heme-1 in the Cyt subunit of the co-complex was detected upon HiPIP binding (Fig. 4c and Supplementary Fig. 6a). Heme distortion has been suggested to modulate various properties of the heme group including its reduction potential. It was demonstrated that decreasing heme distortion resulted in a decrease in the reduction potential^39^. This infers that bending of heme-1 upon HiPIP binding should increase its reduction potential, making it energetically more favorable to accept electrons from HiPIP by reducing the energy gap. However, it should be pointed out that the effect of heme distortion on reduction potential may be less than that induced by electrostatic interactions and/or hydrophobicity around the heme pocket^40^. There is a strong correlation between the reduction potential and the surrounding hydrophobicity: reduction potential increases with decreasing heme exposure to solvent^41^. Based on this result, the reduction potential of heme-1 in the HiPIP:LH1-RC complex is expected to increase because the solvent-exposed edge of heme-1 as seen in Supplementary Fig. 5e, f is covered by the hydrophobic surface of HiPIP, leading to an increased hydrophobicity upon HiPIP binding. This effect again could reduce the energy gap, making the heme-1 more favorable for accepting electrons from HiPIP.

Based on the HiPIP:LH1-RC co-crystal structure determined in this work, Leu63 in *Tch. tepidum* HiPIP is predicted to be the bridging residue for the electron transfer from the 4Fe-4S cluster to heme-1 (Fig. 5b), consistent with the result of early molecular calculations using a docking model^5^. However, despite the high sequence identity (87%; Supplementary Fig. 3) and structural similarity between the *Tch. tepidum* and *Alc. vinosum* HiPIPs, the conserved Phe48 in *Alc. vinosum* HiPIP was predicted to be the mediating residue in the docking model^5^, indicating that the bridging residue could vary between species and that the distance between the donor and acceptor is the main determinant of the rate of electron tunneling^37^. The aromatic ring of Phe48 in *Alc. vinosum* HiPIP was almost parallel to the plane of the heme-1 group^21^; this is in contrast to that in the *Tch. tepidum* HiPIP:LH1-RC structure where the two planes are inclined by 55˚ toward each other (Fig. 2c). Moreover, the contact surface area of the *Tch. tepidum* HiPIP:LH1-RC in our study was found to be significantly smaller than that calculated for the HiPIP-docked RC of *Alc. vinosum*^21^. This points to difficulties in docking-model-based calculations that do not fully account for weak hydrophobic interactions.

In summary, our structural analysis has revealed a critical step of interprotein electron transfer that precedes the intra-protein steps in the purple bacterial photosynthetic RC, and a step that is the rate-determining one in the entire electron transport chain leading to the Q_B_ site. Moreover, the *Tch. tepidum* HiPIP:LH1-RC structure resolves uncertainty over which heme group in the tetraheme Cyt subunit is the direct electron acceptor from the HiPIP and provides a structural snapshot of how this thermodynamically unfavorable interprotein electron transfer occurs. The LH1-bound RC with HiPIP structure also unites light-harvesting, energy transfer, charge separation, and electron transport into one system and therefore provides a more robust model for studying LH-coupled long-range trans-protein electron tunneling processes in photosynthetic organisms.

## Methods

### Co-crystallization of the HiPIP:LH1-RC complex

Purifications of the HiPIP and LH1-RC from *Tch. tepidum* strain MC were conducted as previously described^29,42^. Soluble proteins were separated from membrane fractions by centrifugation of the sonicated cells. The HiPIP was purified by a DEAE column (Toyopearl 650S, TOSOH) with 200 mM NaCl in 20 mM Tris-HCl buffer (pH 8.5). LH1-RC was solubilized from the membrane fraction by two-step detergent treatments (0.3%w/v LDAO and 1%w/v DDM), followed by purification using the same DEAE column with 20 mM Tris-HCl buffer (pH 7.5) containing 0.05 w/v DDM and 50 mM CaCl_2_. Absorption spectra and a co-crystal of the purified proteins are shown in Supplementary Fig. 1. Based on the spectrum, HiPIP was purified in the reduced state. Co-crystallization was carried out using a micro-batch-under-oil method^13^ by mixing HiPIP and LH1-RC in a stoichiometric ratio of 15:1 in a buffer containing 30 mM sodium succinate (pH 5), 20 mM CaCl_2_, 3.4%(w/v) *n*-octyl-phosphocholine (OPC), and 30%(w/v) polyethylene glycol (PEG) 1500. Molar extinction coefficients used for calculations of the protein concentrations were 4322 cm^−1^ M^−1^ for LH1-RC at 915 nm^43^ and 17.3 cm^−^^1^ M^−1^ for HiPIP at 389 nm obtained from inductively coupled plasma atomic emission spectroscopy^44^, which is consistent with the reported value^21^. Concentration of the LH1-RC used for co-crystallization was 0.0694 mM. Binding of the HiPIP to LH1-RC was confirmed by ITC of the individual proteins and by matrix-assisted laser desorption ionization time-of-flight mass spectroscopy (MALDI/TOF-MS) of the dissolved crystals using the methods described elsewhere^44,45^. ITC profile was obtained with titration of 0.134 mM HiPIP in a buffer containing 25 mM CaCl_2_ and 0.05% DDM to 0.0353 mM LH1-RC solution in the same buffer at 25 ˚C. Co-crystals were obtained at 20 °C with suitable sizes (~0.4 × 0.7 × 0.3 mm) after 5 days (Supplementary Fig. 1). Post-crystallization treatment followed the same method as that for the LH1-RC^13^. The co-crystals were transferred to into cryoprotectant solution containing 30 mM sodium succinate (pH 5), 20 mM CaCl_2_, 3.4%w/v OPC, 15%w/v glycerol, and 30%w/v PEG 1500, and flash-frozen immediately in a liquid-nitrogen container.

### Diffraction data collection and structural analysis

X-ray diffraction data were collected at BL41XU and BL44XU of SPring-8 (Hyogo, Japan), and BL1A and BL17A of the Photon Factory (Ibaraki, Japan) at 100 K. The diffraction data were processed, integrated, and scaled using the XDS package^46^. Structure determination was performed by the molecular replacement method using the *Tch. tepidum* LH1-RC structure (PDB ID 5Y5S)^13^ as search model by the Phaser program^47^ in PHENIX^48^. The bound HiPIP structure was manually modeled based on the electron density map using the *Tch. tepidum* HiPIP structure (PDB ID 1EYT, 1.5-Å resolution)^49^ by Coot^50^. The initial HiPIP:LH1-RC model was then refined by *Phenix.refine*^14^. Positional and isotropic displacement parameters were refined in the resolution range of 30.0–2.9 Å. The final model was refined to 2.9 Å resolution with *R*_free_ = 24.8% and *R*_work_ = 21.9% (Supplementary Table 1). Accessible surface areas were estimated using AREAIMOL^51^ in CCP4 with a probe radius of 1.4 Å. Surface charge distributions were calculated using the Adaptive Poisson-Boltzmann Solver software suite^52^ and the PDB2PQR sever (ver. 2.1.1)^53^. The electron transfer pathways from the 4Fe-4S cluster in HiPIP to the special pair BChls in the RC through the tetraheme Cyt subunit were predicted using the *Pathways* plugin^15^ for VMD^54^. Figures were generated with Pymol^55^ unless otherwise stated. Coordinates of the HiPIP:LH1-RC co-crystal structure have been deposited in PDB under accession code 7C52.

### Reporting summary

Further information on research design is available in the Nature Research Reporting Summary linked to this article.

## Supplementary information

Supplementary Information

Peer Review File

Reporting Summary

## Data Availability

Coordinates and structure factors were deposited in the Protein Data Bank under accession code 7C52. All other data are available from the corresponding authors upon reasonable request.

## References

[1] Blankenship, R. E. *Molecular Mechanisms of Photosynthesis* (Blackwell Science, 2002).

[2] Nitschke, W. & Dracheva, S. M. In *Anoxygenic Photosynthetic Bacteria* (eds. Blankenship, R. E., Madigan, M. T. & Bauer, C. E.) 775–805 (Kluwer Academic Publishers, 1995).

[3] Tsukatani Y (2004). Phylogenetic distribution of unusual triheme to tetraheme cytochrome subunit in the reaction center complex of purple bacteria. Photosynth. Res..

[4] van Driessche G (2003). Amino acid sequences and distribution of high-potential iron-sulfur proteins that donate electrons to the photosynthetic reaction center in phototrophic proteobacteria. J. Mol. Evol..

[5] Ciurli S, Musiani F (2005). High potential iron-sulfur proteins and their role as soluble electron carriers in bacterial photosynthesis: tale of a discovery. Photosynth. Res..

[6] Nagashima S, Nagashima KVP (2013). Comparison of photosynthesis gene clusters retrieved from total genome sequences of purple bacteria. Adv. Bot. Res..

[7] Adir N (1996). Co-crystallization and characterization of the photosynthetic reaction center–cytochrome *c*_2_ complex from *Rhodobacter sphaeroides*. Biochemistry.

[8] Axelrod HL (2002). X-ray structure determination of the cytochrome *c*_2_:reaction center electron transfer complex from *Rhodobacter sphaeroides*. J. Mol. Biol..

[9] Verméglio A, Li J, Schoepp-Cothenet B, Pratt N, Knaff DB (2002). The role of high-potential iron sulfur and cytochrome *c*_8_ as alternative electron donors to the reaction center of *Chromatium vinosum*. Biochemistry.

[10] Nagashima KVP, Matsuura K, Shimada K, Verméglio A (2002). High-potential iron-sulfur protein (HiPIP) is the major electron donor to the reaction center complex in photosynthetically growing cells of the purple bacterium *Rubrivivax gelatinosus*. Biochemistry.

[11] Madigan MT (1984). A novel photosynthetic bacterium isolated from a Yellowstone hot spring. Science.

[12] Hirano Y, Takeda K, Miki K (2016). Charge-density analysis of an iron-sulfur protein at an ultra-high resolution of 0.48 Å. Nature.

[13] Yu L-J, Suga M, Wang-Otomo Z-Y, Shen J-R (2018). Structure of photosynthetic LH1-RC supercomplex at 1.9 Å resolution. Nature.

[14] Afonine PV (2012). Towards automated crystallographic structure refinement with phenix.refine. Acta Cryst..

[15] Balabin IA, Hu X, Beratan DN (2012). Exploring biological electron transfer pathway dynamics with the *Pathways* plugin for VMD. J. Comput. Chem..

[16] Pelletier H, Kraut J (1992). Crystal structure of a complex between electron transfer partners, cytochrome c peroxidase and cytochrome c. Science.

[17] Solmaz SRN, Hunte C (2008). Structure of complex III with bound cytochrome *c* in reduced state and definition of minimal core interface for electron transfer. J. Biol. Chem..

[18] Díaz-Moreno I (2014). The dynamic complex of cytochrome *c*_6_ and cytochrome *f* studied with paramagnetic NMR spectroscopy. Biochim. Biophys. Acta Bioenerg..

[19] Terasaka E (2017). Dynamics of nitric oxide controlled by protein complex in bacterial system. Proc. Natl Acad. Sci. USA.

[20] Shimada S (2017). Complex structure of cytochrome *c*–cytochrome *c* oxidase reveals a novel protein–protein mode. EMBO J..

[21] Venturoli G (2004). Electron transfer from HiPIP to the photooxidized tetraheme cytochrome subunit of *Allochromatium vinosum* reaction center: new insights from site-directed mutagenesis and computational studies. Biochemistry.

[22] Osyczka A (1999). Comparison of the binding sites for high-potential iron-sulfur protein and cytochrome *c* on the tetraheme cytochrome subunit bound to the bacterial photosynthetic reaction center. Biochemistry.

[23] Chodera JD, Mobley DL (2013). Entropy–enthalpy compensation: role and ramifications in biomolecular ligand recognition and design.. Annu. Rev. Biophys..

[24] Fox JM, Zhao M, Fink MJ, Kang K, Whitesides GM (2018). The molecular origin of enthalpy/entropy compensation in biomolecular recognition. Annu. Rev. Biophys..

[25] Marco P (2018). Binding of ferredoxin to algal photosystem I involves a single binding site and is composed of two thermodynamically distinct events. Biochim. Biophys. Acta Bioenerg..

[26] Jelesarov I, Bosshard HR (1994). Thermodynamics of ferredoxin binding to ferredoxin:NADP^+^ reductase and the role of water at the complex interface. Biochemistry.

[27] Martínez-Júlvez M, Medina M, Velázquez-Campoy A (2009). Binding thermodynamics of ferredoxin:NADP^+^ reductase: two different protein substrates and one energetics. Biophys. J..

[28] Dey A (2005). Sulfur K-edge XAS and DFT calculations on [Fe_4_S_4_] clusters: effects of H-bonding and structural distortion on covalency and spin topology. Inorg. Chem..

[29] Kobayashi M, Saito T, Takahashi K, Wang Z-Y, Nozawa T (2005). Electronic properties and thermal stability of soluble redox proteins from a thermophilic purple sulfur photosynthetic bacterium, *Thermochromatium tepidum*. Bull. Chem. Soc. Jpn.

[30] Drepper F, Saito T, Kobayashi M, Nozawa T, Mathis P (1998). Electron transfer reactions of high-potential cytochromes in the reaction center of *Chromatium tepidum*. Photosynth. Res..

[31] Chen I-P, Mathis P, Koepke J, Michel H (2000). Uphill electron transfer in the tetraheme cytochrome subunit of the *Rhodopseudomonas viridis* photosynthetic reaction center: evidence from site-directed mutagenesis. Biochemistry.

[32] Alric J (2006). Kinetic performance and energy profile in a roller coaster electron transfer chain: a study of modified tetraheme-reaction center constructs. J. Am. Chem. Soc..

[33] Osyczka A, Nagashima KVP, Shimada K, Matsuura K (1999). Interaction site for high-potential iron-sulfur protein on the tetraheme cytochrome subunit bound to the photosynthetic reaction center of *Rubrivivax gelatinosus*. Biochemistry.

[34] Knaff DB (1991). Reaction of cytochrome *c*_2_ with photosynthetic reaction centers from *Rhodopseudomonas viridis*. Biochemistry.

[35] Osyczka A (1998). Interaction site for soluble cytochromes on the tetraheme cytochrome subunit bound to the bacterial photosynthetic reaction center mapped by site-directed mutagenesis. Biochemistry.

[36] Ortega JM, Drepper F, Mathis P (1999). Electron transfer between cytochrome *c*_2_ and the tetraheme cytochrome *c* in *Rhodopseudomonas viridis*. Photosynth. Res..

[37] Page CC, Moser CC, Chen X, Dutton PL (1999). Natural engineering principles of electron tunneling in biological oxidation-reduction. Nature.

[38] Nitschke W, Jubault-Bregler M, Rutherford AW (1993). The reaction center associated tetraheme cytochrome subunit from *Chromatium vinosum* revisited: a reexamination of its EPS properties. Biochemistry.

[39] Olea J (2008). Probing the function of heme distortion in the H-NOX family. ACS Chem. Biol..

[40] Saito K (2012). Deformation of chlorin rings in the photosystem II crystal structure. Biochemistry.

[41] Tezcan FA, Winkler JR, Gray HB (1998). Effects of ligation and folding on reduction potentials of heme proteins. J. Am. Chem. Soc..

[42] Suzuki H (2007). Purification, characterization and crystallization of the core complex from thermophilic purple sulfur bacterium *Thermochromatium tepidum*. Biochim. Biophs. Acta Bioenerg..

[43] Nagatsuma S (2019). Phospholipid distributions in purple phototrophic bacteria and LH1-RC core complexes. Biochim. Biophys. Acta Bioenerg..

[44] Kimura Y (2017). Effects of calcium ions on the thermostability and spectroscopic properties of the LH1-RC complex from a new thermophilic purple bacterium *Allochromatium tepidum*. J. Phys. Chem. B.

[45] Kimura Y (2018). C-terminal cleavage of the LH1 α-polypeptide in the Sr^2+^-cultured *Thermochromatium tepidum*. Photosynth. Res..

[46] Kabsch W (2010). XDS. Acta Cryst..

[47] McCoy AJ (2007). Phaser crystallographic software. J. Appl. Cryst..

[48] Adams PD (2010). PHENIX: a comprehensive Python-based system for macromolecular structure solution. Acta Cryst..

[49] Nogi T, Fathir I, Kobayashi M, Nozawa T, Miki K (2000). Crystal structures of photosynthetic reaction center and high-potential iron-sulfur protein from *Thermochromatium tepidum*: thermostability and electron transfer. Proc. Natl Acad. Sci. USA.

[50] Emsley P, Lohkamp B, Scott WG, Cowtan K (2010). Features and development of *Coot*. Acta Cryst..

[51] Lee B, Richards FM (1971). The interpretation of protein structures: Estimation of static accessibility. J. Mol. Biol..

[52] Jurrus E (2018). Improvements to the APBS biomolecular solvation software suite. Protein Sci..

[53] Dolinsky TJ, Nielsen JE, McCammon JA, Baker NA (2004). PDB2PQR: an automated pipeline for the setup of Poisson-Boltzmann electrostatic calculations. Nucleic Acids Res..

[54] Humphrey W, Dalke A, Schulten K (1996). VMD: visual molecular dynamics. J. Mol. Graph..

[55] DeLano, W. L. *The PyMOL Molecular Graphics System* (DeLano Scientific, LCC, 2004).

