# Supplementary material for: Crystal structure of a photosynthetic LH1-RC in complex with its electron donor HiPIP
Source: Nat Commun. 2021 Feb 17;12:1104. doi: 10.1038/s41467-021-21397-9 (PMC7889895; doi:10.1038/s41467-021-21397-9)
Supplement: Supplementary file 1 — Supplementary Information [file 41467_2021_21397_MOESM1_ESM.pdf]

SUPPLEMENTARY INFORMATION

Crystal Structure of a Photosynthetic LH1-RC in Complex with its Electron Donor HiPIP

T. Kawakami, L.-J. Yu, et al.

Supplementary Table 1 Data collection and refinement statistics.

Diffraction data	
Space group	$P2_1$
Cell dimensions (Å)	a = 96.4, b = 183.3, c = 123.9, β=112.6°
Wavelength (Å)	0.98
Resolution (Å)	48.220 – 2.893 (2.996 – 2.893)
Multiplicity	6.5 (5.5)
R-merge	0.127 (2.06)
$I / \sigma (I)$	10.3 (0.69)
Completeness (%)	97.4 (87.7)
Wilson *B*-factor	103.66
$CC_{1/2}$	0.998 (0.334)
ISa	16.7
Refinement	
Resolution (Å)	29.99 – 2.893 (2.996 – 2.893)
No. Reflections	86258 (6927)
R-work	0.2188 (0.3729)
R-free	0.2477 (0.4015)
No. of non-hydrogen atoms	27405
macromolecules	22607
ligands	4798
Protein residues	2890
Average *B*-factor ($Å^2$)	128.67
macromolecules	128.05
ligands	131.61
RMS (bonds (Å) / angles (°))	0.008 / 1.27
Ramachandran plot (%)	
favored / allowed / outlier	98.5 / 1.39 / 0.11
Rotamer outliers (%)	1

Values in parentheses represent the highest-resolution shell.

Supplementary Table 2 Protein-protein distances between HiPIP and Cyt-subunit within 4.0 Å.

HiPIP	Cyt-subunit	Distance (Å)
**Thr79 Oγ**	**Trp116 Nε1**	**2.3** (H-bond)
Thr13 Oγ1	Val95 Cγ1	3.7
Thr13 Cγ2	Val95 Cγ1	3.9
Ala16 Cβ	Pro103 O*	3.4
Arg33 Nε	Asn108 Oδ1	3.1 (H-bond)
Arg33 Nη2	Asn108 Oδ1	3.6 (H-bond)
Arg33 Nη2	Asn108 Nδ2	3.8 (H-bond)
Pro34 Cγ	Pro113 O*	3.2
Pro34 Cγ	Pro113 Cβ	3.8
Phe48 Cζ	Trp116 Nε1	3.8
Phe48 Cζ	heme-1 $C2^1$	3.8
Gln62 Nε2	Val87 Cγ1	3.4
Gln62 Oε1	Tyr76 Oη	3.6 (H-bond)
Gln62 O*	Thr91 Oγ	3.6 (H-bond)
Leu63 O*	Val95 Cγ2	3.3
Leu63 Cδ2	heme-1 $C2^1$	3.5
Pro65 O*	Pro63 Cδ	3.2
Pro65 Cβ	Pro63 O*	3.3
Pro65 Cβ	Ala65 Cβ	3.5
Pro65 Cγ	Arg92 Cγ	3.9
Lys67 Cε	Pro63 Cγ	3.8
Ser77 Oγ	Trp116 Cζ2	3.5
Ser77 Oγ	heme-1 $C2^1$	3.5

* main-chain carbonyl oxygen.

Supplementary Table 3 Coupling strengths between the donor-acceptor pairs calculated by the *Pathways* plugin for VMD using default parameters.

From	To	Distance (Å)	Coupling strength ($\times 10^{-5}$)
**S1(4Fe-4S)**	**Fe(heme-1)**	**12.2**	**1.65**[a]
S2(4Fe-4S)	Fe(heme-1)	15.2	0.62
S3(4Fe-4S)	Fe(heme-1)	14.8	0.62
S4(4Fe-4S)	Fe(heme-1)	15.3	0.59
**Fe4(4Fe-4S)**	**Fe(heme-1)**	**13.7**	**1.04**
Fe2(4Fe-4S)	Fe(heme-1)	13.7	0.99
Fe3(4Fe-4S)	Fe(heme-1)	14.0	0.99
Fe1(4Fe-4S)	Fe(heme-1)	15.9	0.37
**Fe(heme-1)**	**Fe(heme-2)**	**13.9**	**6.97** (Path 1)[b]
			6.32 (Path 2)
			5.77 (Path 3)
**Fe(heme-2)**	**Fe(heme-4)**	**16.2**	**10.4** (Path 1)
			7.91 (Path 2)
			7.24 (Path 3)
**Fe(heme-4)**	**Fe(heme-3)**	**13.9**	**7.57** (Path 1)
			4.54 (Path 2)
			4.54 (Path 3)
**Fe(heme-3)**	**Mg(BChl *a*, L)**	**20.3**	**0.038** (Path 1)
			0.026 (Path 2)
			0.026 (Path 3)
**Fe(heme-3)**	**Mg(BChl *a*, M)**	**20.6**	**0.027** (Path 1)
			0.023 (Path 2)
			0.016 (Path 3)

a: The bold fonts indicate the most probable pathway with maximum coupling strengths.
b: The pathway numbers correspond to the strengths indicated in Supplementary Fig. 7.

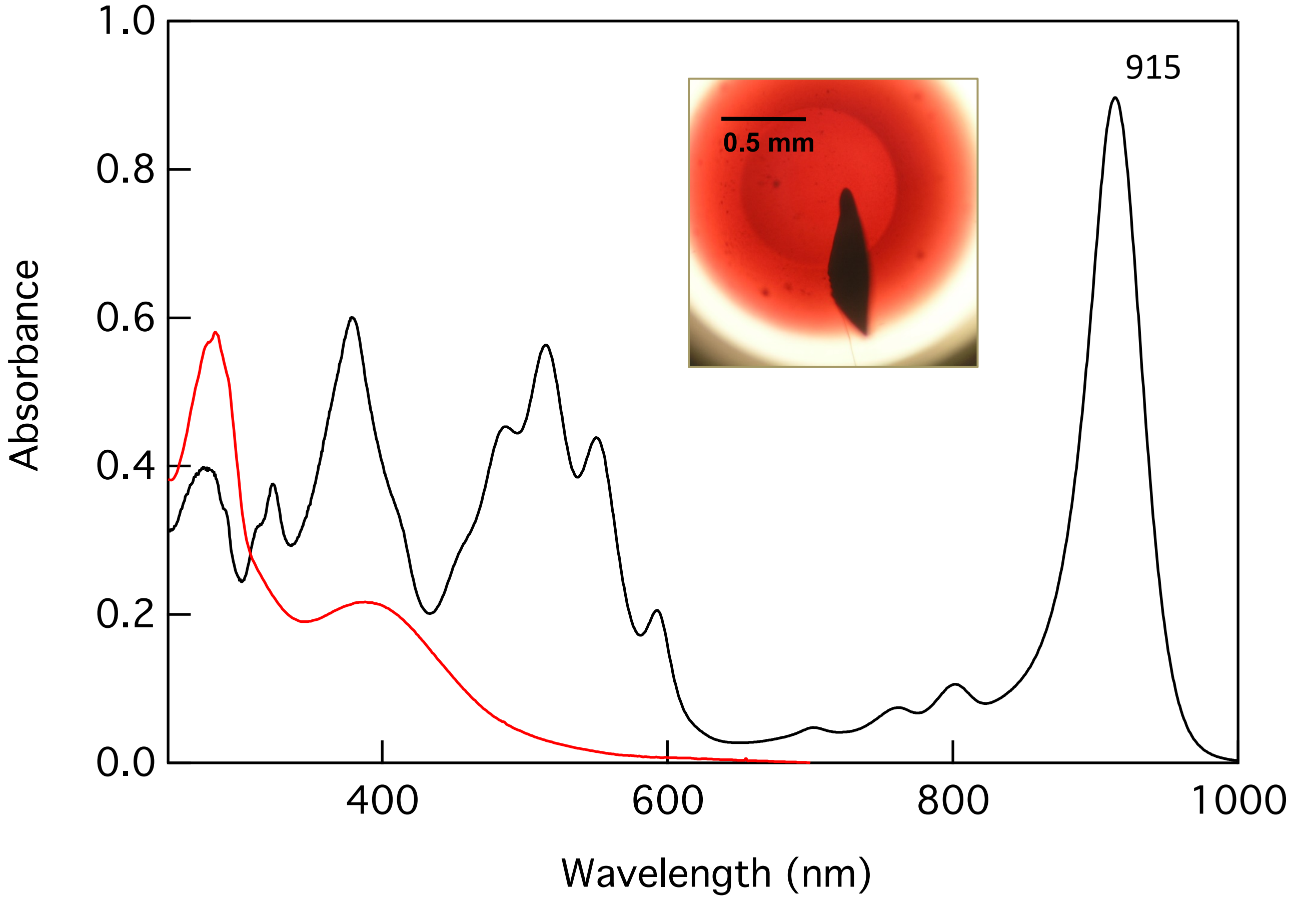

Supplementary Figure 1 Absorption spectra of the purified HiPIP (red line) and LH1-RC (black line). Inset shows a HiPIP:LH1-RC co-crystal formed at 20 °C after 5 days.

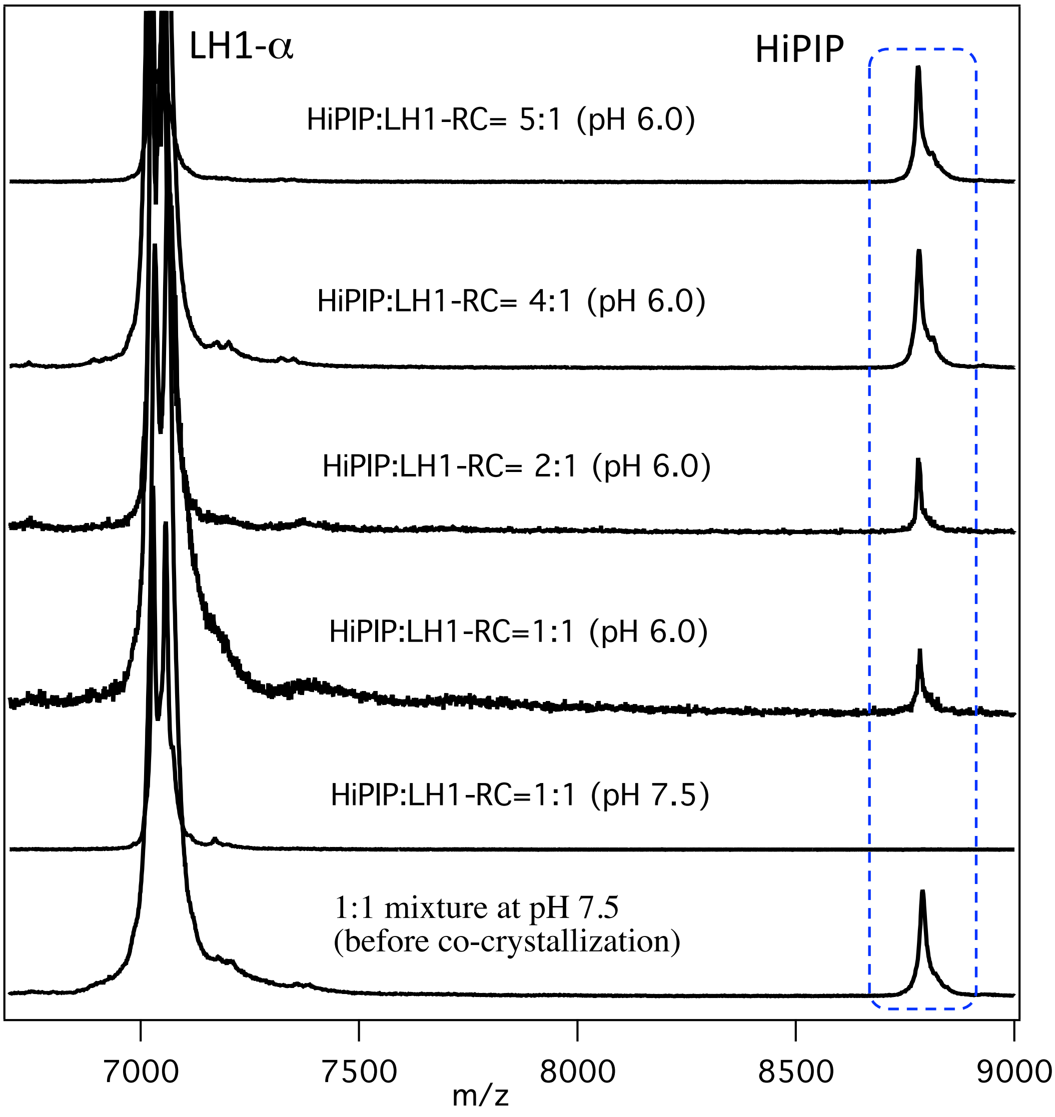

Supplementary Figure 2 MALDI/TOF-MS spectra of the HiPIP:LH1-RC mixture (bottom) and dissolved crystals (other curves) formed at different molar ratios and pH. The signals of HiPIP are shown in the blue dashed box.

Cyt-subunit

```
                        60        70        80        90       100       110       120
Tch. tepidum (γ)        ESLPAADSTGPKASEVYQNVQVLKDLSVGEFTRTMVAVTTWVSPKEGCNYCHVPGNWASDD
Alc. vinosum (γ)        ESLPAAAPGGPSVSDVYENVQVLKDLSVAEFTRTMVAVTTWVAPKEGCNYCHVPGNWASDD
Ru. gelatinosus (β)     VATPVADDSGPRANQVFQNVKVLGHLSVAEFTRQMAAITEWVSPTEGCNYCHTEN-LADDS
Af. marina (α)          DPFPAVSAEGPRASEVYENVHVLGDLSEEQFLRVMTVITEWVSPEQGCAYCHDENDLAAER
                           *..   ** ..:*::**:** .**  :* * *..:* **:* :** ***  .  * :
                                                                       Heme-1
```

HiPIP

```
                          10        20        30        40        50           60        70        80
Tch. tepidum      AAPANAVTADDPTAIALKYNQDATKSERVAAARPGLPPEEQHCANCQFMQANVG--EGDWKGCQLFPGKLINVNGWCASWTLKAG
Alc. vinosum      SAPANAVAADDATAIALKYNQDATKSERVAAARPGLPPEEQHCANCQFMQADAAGATDEWKGCQLFPGKLINVNGWCASWTLKAG
Ru. gelatinosus   SAMAAPVDEKDPQAVALGYVADAAKADKAKYKQFVAG---SHCGNCALFQGKPT---DATGNCPLFAGKQVNNKGWCSAWAKKA-
Af. marina        GSANAQIANKAKQAIAGYQTTPKGRQE---------------CDNCRHFQAPDA--------CKVVQG-TISPKGWCRLYAKK--
                  .:    :  .   *:*       : :                * **  :*.           * :. *  :. :***  :: *
```

Supplementary Figure 3 Comparisons of the partial amino acid sequence of C-subunit (top) and the sequence of HiPIP (bottom) from *Tch. tepidum* (γ-class) with those from other purple bacteria: *Allochromatium vinosum* (γ-class), *Rubrivivax gelatinosus* (β-class) and *Afifella marina* (α-class, formerly *Rhodobium marinum*). Symbol scheme: (*) identical, (.) similar, (:) highly similar. Bold magenta letters represent interacting residues between the two proteins of *Tch. tepidum* with distances shorter than 4.0 Å (see Supplementary Table 1).

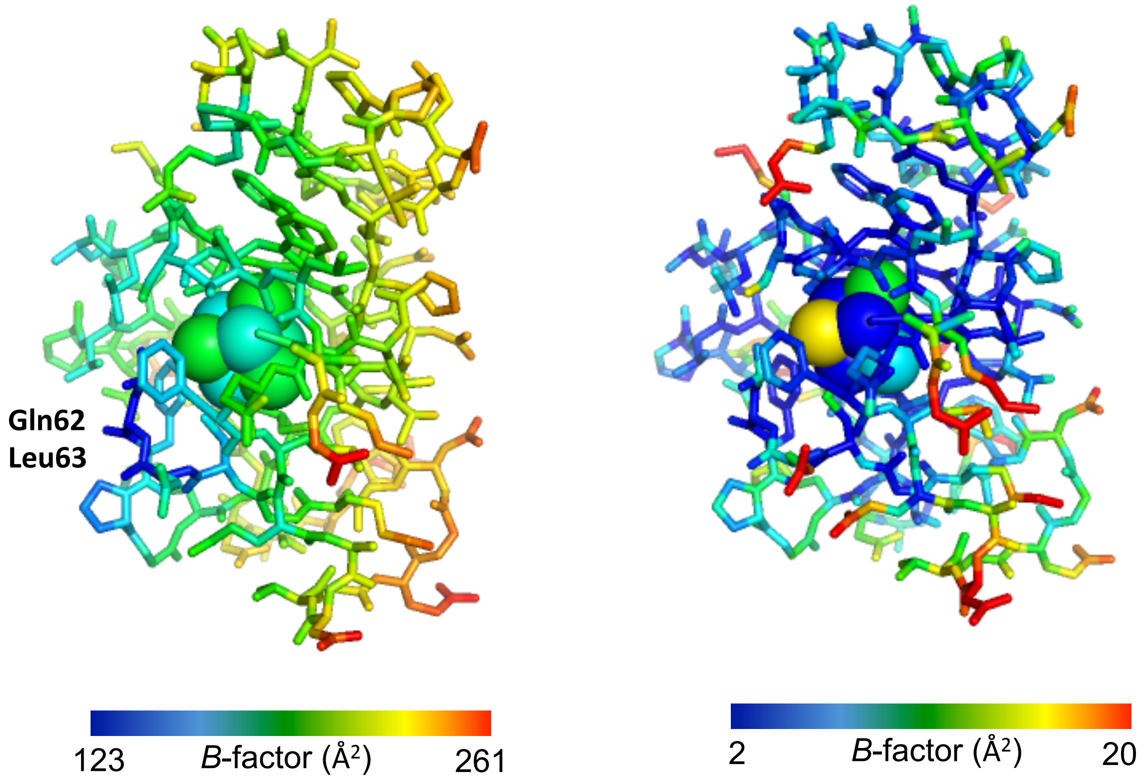

Supplementary Figure 4 Comparison of the *B*-factor distributions between the bound (*left*) and free (*right*, PDB ID: 1EYT) HiPIPs. Colors represent values of *B*-factor as indicated in the color bar.

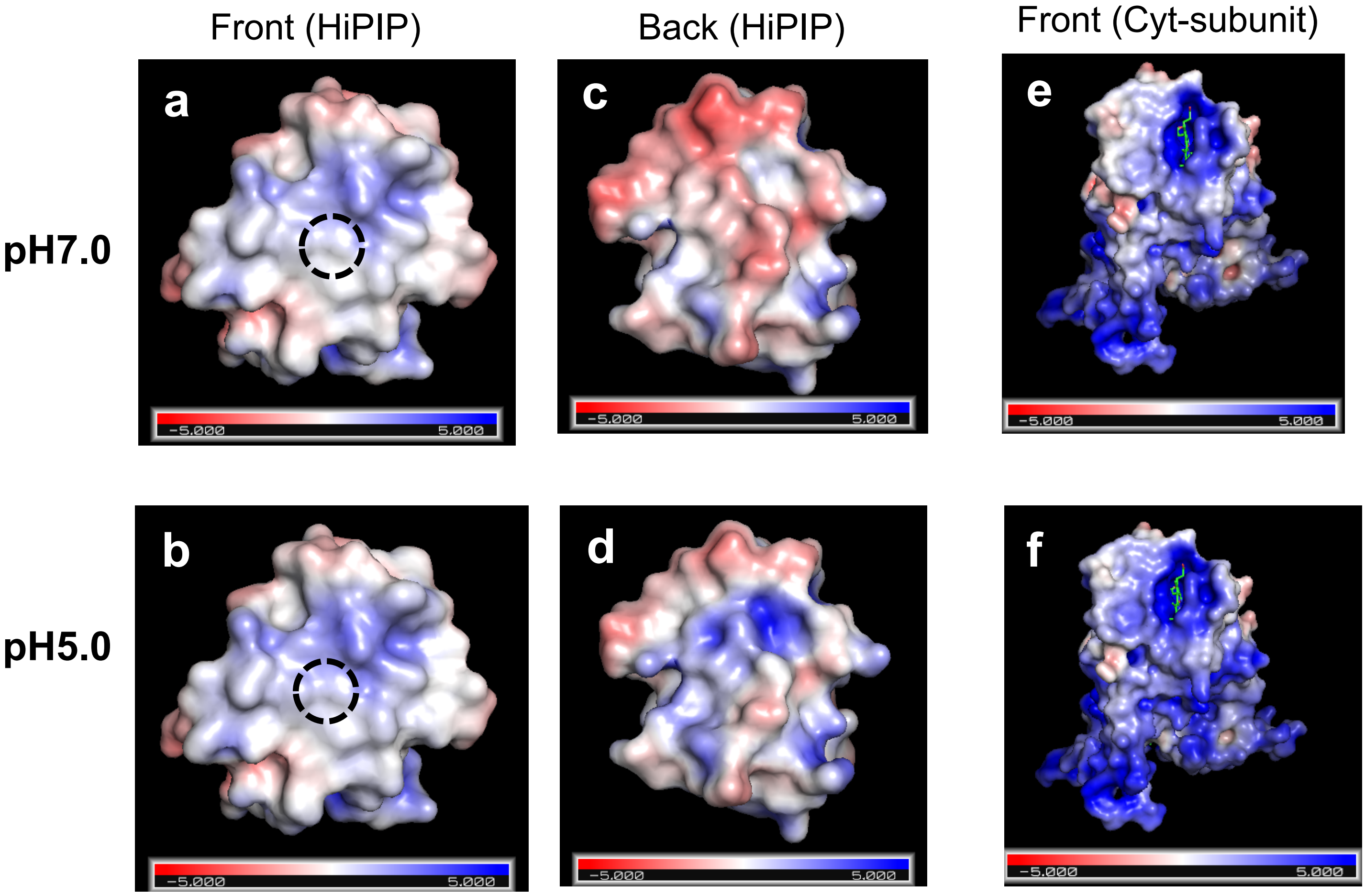

Supplementary Figure 5 Surface charge distributions of the binding surface (front) of HiPIP (a, b) and Cyt-subunit (e, f), and opposite side (back) of the binding surface of HiPIP (c, d) at pH7.0 (a, c, e) and pH5.0 (b, d, f). The distributions are color-coded according to the electrostatic potential from -5.0 k_BT (red, negative charge) to +5.0 k_BT (blue, positive charge) in the scale bars. Black dotted circles indicate the position of 4Fe-4S cluster inside HiPIP. The heme-1 in Cyt-subunit is shown by green sticks.

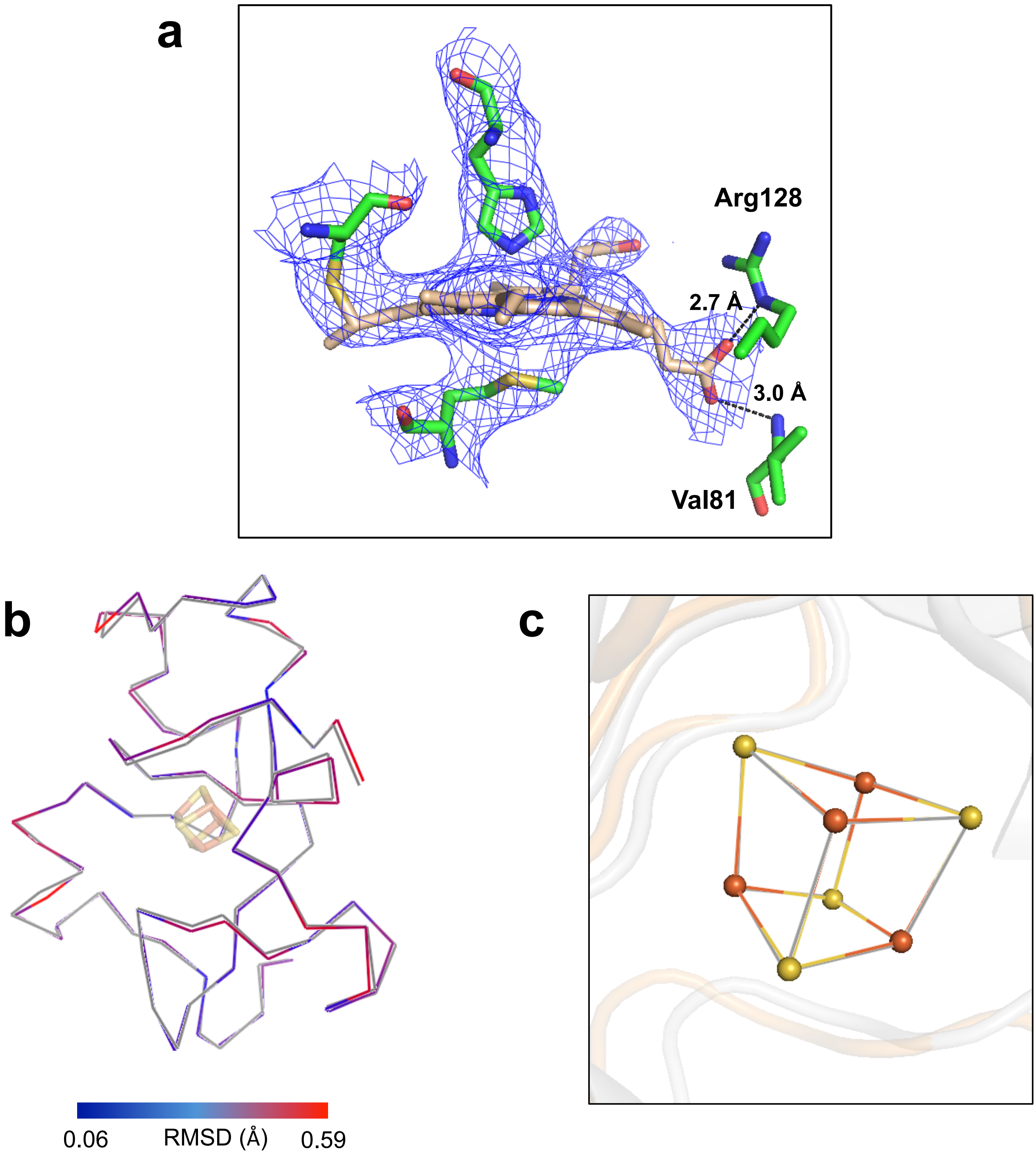

Supplementary Figure 6 (a) Electron density map (2mFo–DFc) for the heme-1 group and some surrounding residues in the Cyt-subunit at 0.92σ. **(b)** Superposition of the Cα carbons of the HiPIP (colored) in co-complex with those in free HiPIP (grey, PDB ID: 1EYT). The Cα traces are color-coded by the values of RMSD as indicated in the color bar. **(c)** Superposition of the sulfur and Fe atoms of the 4Fe-4S cluster (colored) in co-complex with those in free HiPIP (grey, PDB ID: 1EYT).

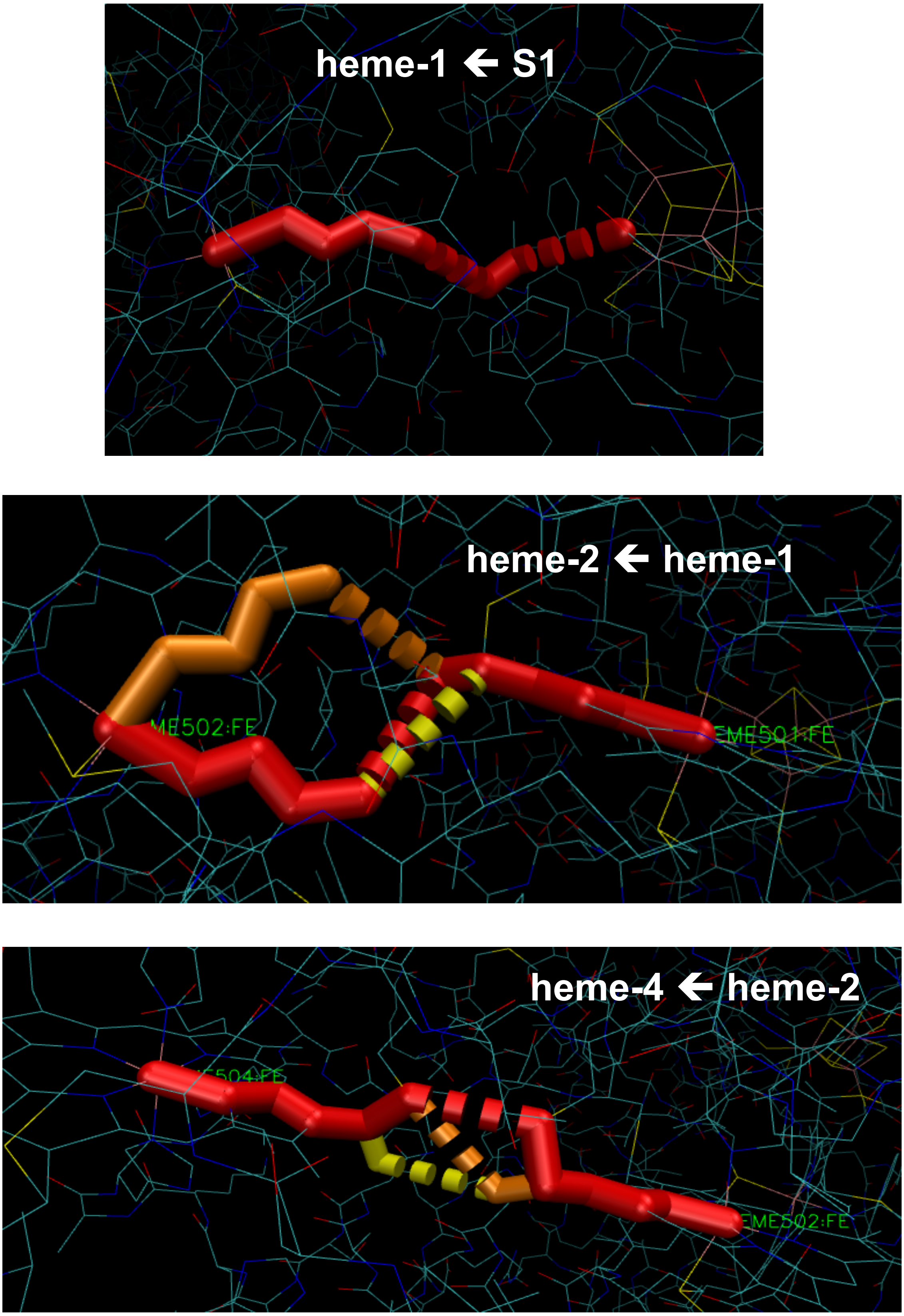

Supplementary Figure 7 (combined with next page).

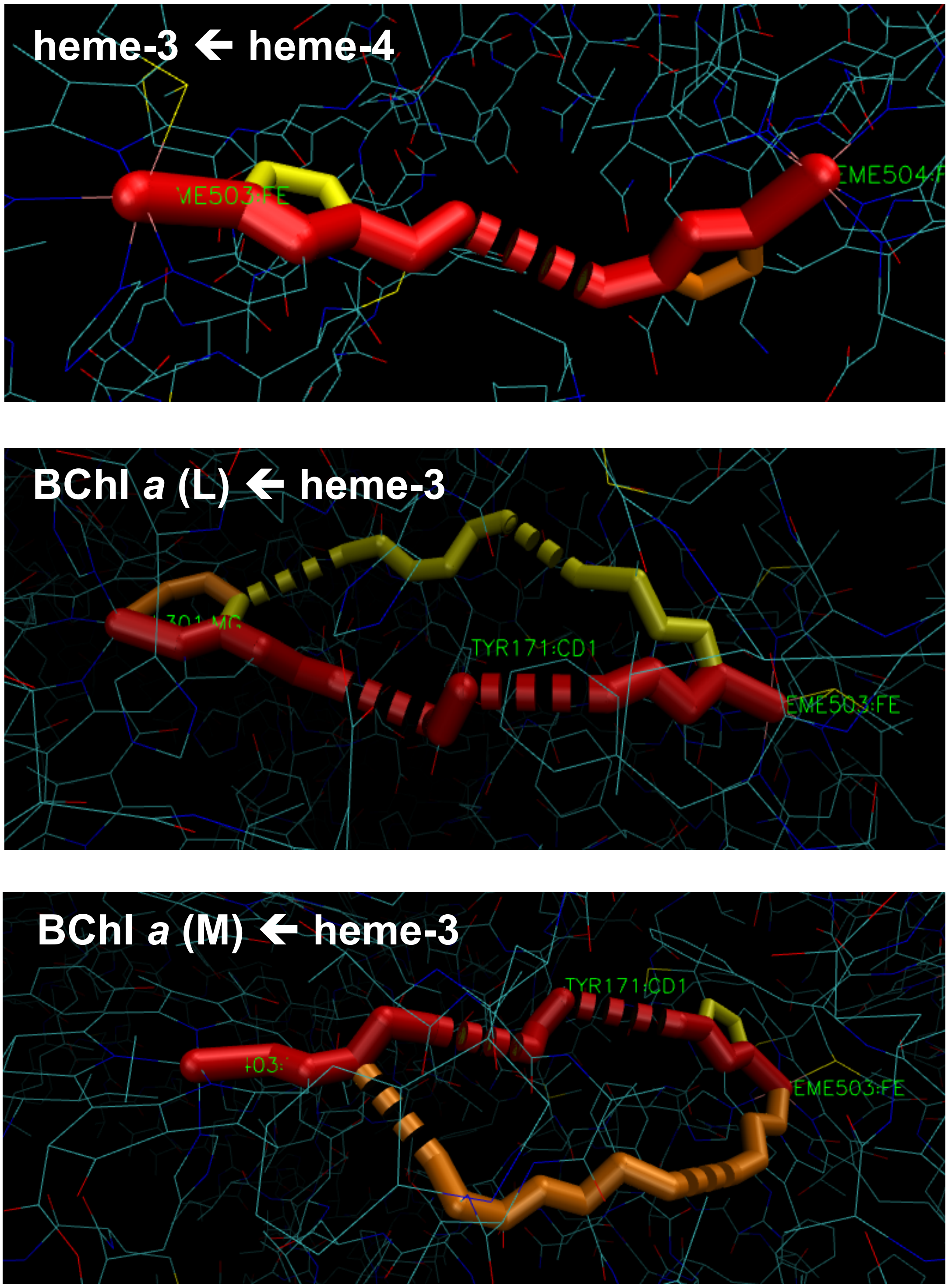

Supplementary Figure 7 (continued from last page) Stepwise electron transfer pathways for each donor-acceptor pair predicted by the *Pathways* plugin for VMD using standard parameters. Solid cylinders indicate pathway steps mediated by covalent bonds, and dashed cylinders indicate through-space jumps or hydrogen bonds. The cylinder diameters indicate the pathway's strength that are color-coded in an order of red (strong), orange and yellow (weak). The figures were generated by the *Pathways* plugin for VMD.

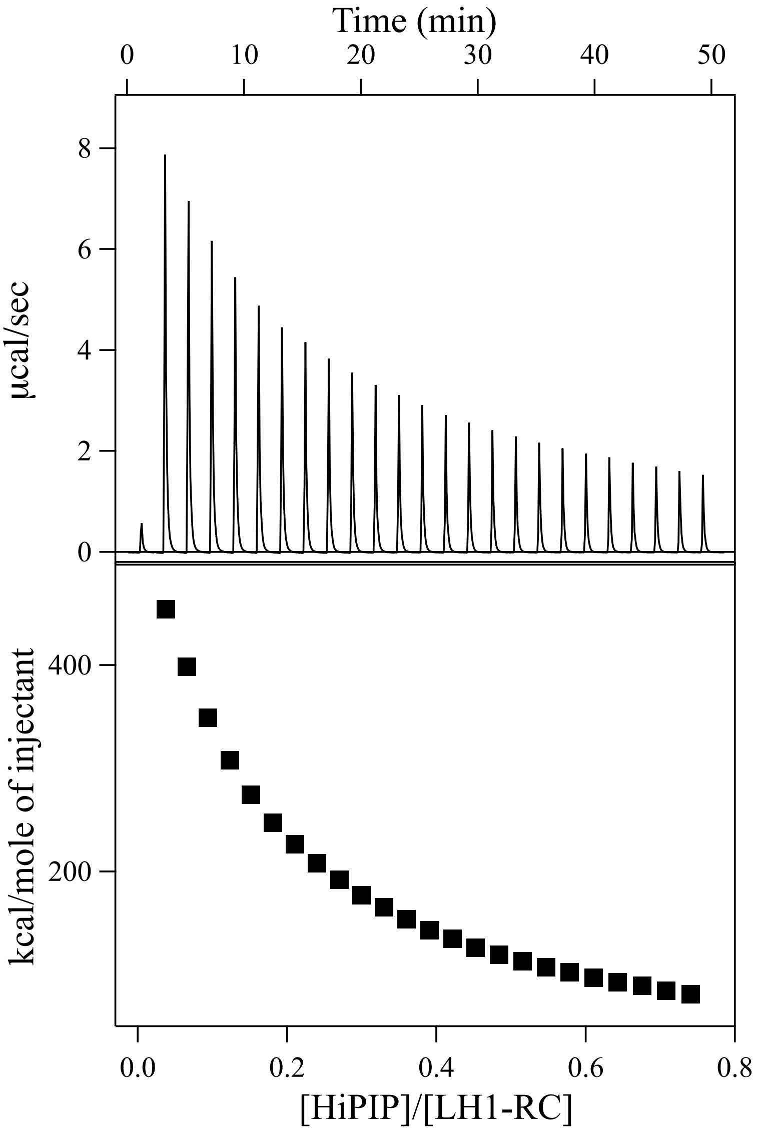

Supplementary Figure 8 ITC measurements. *Top*: ITC profile obtained with titration of 0.134 mM HiPIP in a buffer containing 25 mM $CaCl_2$ and 0.05% DDM to 0.0353 mM LH1-RC solution in the same buffer at 25°C. *Bottom*: Integrated heat per mole of the injectant against molar ratio of HiPIP to LH1-RC.
