# Supplementary material for: Crystal structure of a photosynthetic LH1-RC in complex with its electron donor HiPIP
Source: Nat Commun. 2021 Feb 17;12:1104. doi: 10.1038/s41467-021-21397-9 (PMC7889895; doi:10.1038/s41467-021-21397-9)
Supplement: Supplementary file 3 — Reporting Summary [file 41467_2021_21397_MOESM3_ESM.pdf]

nature research

Corresponding author(s): Z.-Y. Wang-Otomo

Last updated by author(s): Jan 20, 2021

Reporting Summary

Nature Research wishes to improve the reproducibility of the work that we publish. This form provides structure for consistency and transparency in reporting. For further information on Nature Research policies, see our Editorial Policies and the Editorial Policy Checklist.

Statistics

For all statistical analyses, confirm that the following items are present in the figure legend, table legend, main text, or Methods section.

n/a	Confirmed	
☐	☒	The exact sample size (*n*) for each experimental group/condition, given as a discrete number and unit of measurement
☐	☒	A statement on whether measurements were taken from distinct samples or whether the same sample was measured repeatedly
☒	☐	The statistical test(s) used AND whether they are one- or two-sided<br>*Only common tests should be described solely by name; describe more complex techniques in the Methods section.*
☒	☐	A description of all covariates tested
☒	☐	A description of any assumptions or corrections, such as tests of normality and adjustment for multiple comparisons
☒	☐	A full description of the statistical parameters including central tendency (e.g. means) or other basic estimates (e.g. regression coefficient) AND variation (e.g. standard deviation) or associated estimates of uncertainty (e.g. confidence intervals)
☒	☐	For null hypothesis testing, the test statistic (e.g. *F*, *t*, *r*) with confidence intervals, effect sizes, degrees of freedom and *P* value noted<br>*Give P values as exact values whenever suitable.*
☒	☐	For Bayesian analysis, information on the choice of priors and Markov chain Monte Carlo settings
☒	☐	For hierarchical and complex designs, identification of the appropriate level for tests and full reporting of outcomes
☒	☐	Estimates of effect sizes (e.g. Cohen's *d*, Pearson's *r*), indicating how they were calculated

Our web collection on statistics for biologists contains articles on many of the points above.

Software and code

Policy information about availability of computer code

Data collection	UGUI (The data collection software at PF Beamline)
Data analysis	XDS(May 1, 2016), Phenix(1.17.1_3660), Coot0.8.9, Pymol2.3.5, PDB2PQR(2.1.1), CCP4(7.0.078), VMD1.9.3, Pathways plugin1.2

For manuscripts utilizing custom algorithms or software that are central to the research but not yet described in published literature, software must be made available to editors and reviewers. We strongly encourage code deposition in a community repository (e.g. GitHub). See the Nature Research guidelines for submitting code & software for further information.

Data

Policy information about availability of data

All manuscripts must include a data availability statement. This statement should provide the following information, where applicable:

- Accession codes, unique identifiers, or web links for publicly available datasets
- A list of figures that have associated raw data
- A description of any restrictions on data availability

Coordinates of the structure have been deposited in PDB under accession code 7C52. All data and biological materials reported in this study can be provided by the corresponding authors upon reasonable request.

Field-specific reporting

Life sciences study design

All studies must disclose on these points even when the disclosure is negative.

Sample size	No sample size calculation was performed. The co-crystals obtained were reproducible, and the diffraction data were processed with standard crystallographic software (Methods section), which resulted in standard data statistics as shown in Supplementary Table 1.
Data exclusions	No data was excluded
Replication	All replication was successful. Many co-crystals were tested which yielded diffraction data comparable to the one used in the structural analysis of the present study. Among these, diffraction data collected from a typical co-crystal was shown in Supplementary Table 1.
Randomization	The co-crystal used for the structural analysis was selected based on its diffraction quality; other crystals gave rise to similar quality, and the selection was made randomly actually.
Blinding	We checked the quality of the co-crystals based on the diffraction data. The investigators were blinded to group allocation during data collection and analysis.

Reporting for specific materials, systems and methods

We require information from authors about some types of materials, experimental systems and methods used in many studies. Here, indicate whether each material, system or method listed is relevant to your study. If you are not sure if a list item applies to your research, read the appropriate section before selecting a response.

Materials & experimental systems

n/a	Involved in the study
☒	☐ Antibodies
☒	☐ Eukaryotic cell lines
☒	☐ Palaeontology and archaeology
☒	☐ Animals and other organisms
☒	☐ Human research participants
☒	☐ Clinical data
☒	☐ Dual use research of concern

Methods

n/a	Involved in the study
☒	☐ ChIP-seq
☒	☐ Flow cytometry
☒	☐ MRI-based neuroimaging
