# Supplementary material for: Crystal structure of a photosynthetic LH1-RC in complex with its electron donor HiPIP
Source: Nat Commun. 2021 Feb 17;12:1104. doi: 10.1038/s41467-021-21397-9 (PMC7889895; doi:10.1038/s41467-021-21397-9)
Supplement: Supplementary file 2 — Peer Review File [file 41467_2021_21397_MOESM2_ESM.pdf]

REVIEWER COMMENTS

Reviewer #1 (Remarks to the Author):

Summary

This paper is clearly written and it has excellent figures. It reports an interesting advance in the study of the supply of electrons to LH1-RC complexes with bound, four-heme cytochromes.

1. The title states that this co-structure reveals a mechanism for energetically "uphill" interprotein electron tunnelling, but it isn't clear what this mechanism is, and I wonder if 'mechanism' might be overstating things, particularly if the authors mean something involving Leu63. Perhaps 'route' or pathway' might be better. 'Mechanism' might imply a study with site directed mutants, mutant structures and extensive kinetics, for example.

2. Page 4. Results, and also Methods. The co-crystallisation conditions don't specify the redox state of the HiPIP, but presumably it is in the reduced form.

3. Page 4. The call to Figure S3 on page 4 does not mention the comparison with the HiPIP in other species shown in Fig S3, but the existence of the alignments could be flagged up here.

4. Page 7 - Predicted Electron Transfer Pathway. The authors propose that electron transfer from 4Fe-4S to heme-1 is mediated by the sidechain of Leu63 in HiPIP. The authors could comment on how well conserved this residue is and point the reader to supplementary Figure S3. There is nothing noteworthy about Leu63, and presumably any residue in this position would play the same role, by filling the intervening space; Leu63 does not physically accept and donate electrons. The Dutton-Moser 'ruler' (ref 36) shows that the distance between the donor and acceptor is the main determinant of the rate of electron tunnelling, and as long as the distance requirement is satisfied, as it is here, the rate of ET will be adequate. As the authors point out, the measured ET rate for the Tch. tepidum HiPIP/RC pair is much slower than calculated because formation (and likely dissociation) of the ET complex is rate limiting. Thus, Leu63 is not functionally important, and certainly not physiologically so.

5. The lines in figure S7 for the protein are very difficult to see. It may be worthwhile to use a white background and/or increase the line width in this figure.

6. Page 9. The authors describe ITC experiments in which HiPIP was titrated to a molar ratio of 0.8, much lower than the 15:1 used in the study. Did the authors see any interesting behaviour at higher molar ratios and could an affinity be determined from the data? If so how does the affinity compare to previous studies?

7. The discussion is focused on the uphill energy transfer from HiPIP to Heme-1 and quotes potentials from previous studies. Have the authors considered whether bending of Heme-1 in the RC-HiPIP complex may shift the reduction potential of Heme-1 and make the electron transfer from HiPIP more energetically favourable?

Minor corrections:

Abstract: "Structure of the co-complex" to "The structure of the co-complex"
Abstract: "RC in close vicinity" to "RC in close proximity"
Page 3: "group of soluble proteins that shuttle between the RC and…" to "group of soluble proteins that shuttle electrons between the RC and…"
Page 4: "Our structural results here verify…" to "Our structural results verify…"
Page 4: "…and HiPIP but go…" to "…and HiPIP and go…"
Page 4 "…from Glu60 to Asp120 and in close vicinity…" to "…from Glu60 to Asp120 and in close

proximity…”
Page 5 "…exposed to the solvents." to "Exposed to the solvent"
Page 9. Abbreviation ITC is not defined
Fig 1 legend. A in BChl a should be italicised
Fig 4 legend 'room-mean' to 'root-mean'

Reviewer #2 (Remarks to the Author):

The topic of the article is relevant and is of interest to the researchers in the area of bacterial photosynthesis as well as to anyone interested in biological electron transport. The undoubted achievement of this work is the successful crystallization of the short-lived complex HiPIP:LH1-RC and the resolving of its structure with relatively high resolution 2.9 Å. All main conclusions of the manuscript are based on this structure. My main questions, as a reviewer, are also related to the structure.
To start with, authors pay special attention to the B-factor, and it is used to color Figs. 1b and S4. However its value in the HiPIP:LH1-RC structure varies from 123 to 261 and seems to be extremely high for such a resolution. At the same time, too low values of R-factors are noted, which may be related to inaccurate B-factor refinement. The fact that the atoms inside of a protein have lower B-factor (since their positions are more stable) compared to the atoms on the surface of a protein, is known and, to my opinion, does not deserve special attention.
Further, when specifying distances between atoms in the structure, coordinate error for this resolution are usually shown. In your manuscript, they are mentioned neither in the text, nor in the figures and tables. I can offer a reference on the topic - A.A.Vaguine, J. Richelle, S.J.Wodak, SFCHECK: a unified set of procedures for evaluating the quality of macromolecular structure-factor data and their agreement with the atomic model, Acta Crystallogr. D: Biol. Crystallogr. 55 (1999) 191–205. One can expect that the coordinate error at resolution 2.9 Å may be quite large, perhaps at least 0.27-0.29 Å.

I believe that it is hardly correct to make superposition using structures with so different resolutions, 2.9 Å for HiPIP:LH1-RC complex and 1.5 Å for HiPIP protein or 1.9 Å for LH1-RC complex (Figs 4 and S6). It can be noticed that the deviations found in the positions of prosthetic groups or protein are pretty close to the value of the expected coordinate error of the HiPIP:LH1-RC structure.
Thus, most of the conclusions about hydrogen-bonds, distances between amino acid residues, deviations of prosthetic groups and the protein of the cytochrome subunit do not seem very convincing and need to be clarified.
I also have small remarks. Figure 1 is hard to read with too many colors. Similar colors were selected to represent different objects (for example, purple and magenta, Fe-S - yellow-orange ball; Ca - orange ball). Table S1 is shown twice, on the pages 23 and 37.

Reviewer #3 (Remarks to the Author):

In this study a crystal structure at a resolution of 2.9 Å was determined of a complex (contact surface 617 Å2) between the high potential iron-sulfur protein (HiPiP) and the reaction center (RC) isolated from the thermophilic purple sulfur bacterium Tch. Tepidum. Optimum/successful crystallization conditions required an excess of HiPiP, and an HiPiP: RC ratio of approximately 15 at pH 5.
This is an interesting study, but the text has a number of incorrect/questionable statements. Chronologically:
1. p7, l6. Vague-poor form for referencing: "…dominated by the 4Fe-4S cluster (see later)." What does "later" mean?
2. Errata: (1) Results, l3: Fig. S2 not S1, S2.; (2) p7, l6. "…superimposing the…?"; (3); p7, l9: "…binding of ferredoxin."
3. p10. What is the reference for the statement about a '0.7 eV reorganization energy? A formula for the rate of electron transfer and its parameter dependence is not provided.

4. In the manuscript, why are manuscript references 1-37 separated from refs 38-52.
5. Fig. 4C. The colors should be changed so that the contrast between the 2 conformations of the heme can be seen.
6. Fig. 5a is perhaps the most important figure in the manuscript as it relates to the major heading of the manuscript, an extended electron transfer pathway However, one cannot appreciate it because there is no information provided on the distances between the redox centers nor on the redox potentials of the Centers.
With this limited information and none in particular on the redox potentials of the carriers in Fig. 5a, nor on the distances between them it is not clear how this pathway can be characterized as "energetically 'uphill.'"
7. Similarly, it is not clear that there is enough information in Fig. S7 to allow appreciation of nuances of electron transfer in this study. Thus, in its present form, the manuscript is not acceptable for publication.

8. The manuscript needs a Table of Abbreviations.

Response to reviewers:

Reviewer #1

Reviewer #1's comments: Point 1

Summary

This paper is clearly written and it has excellent figures. It reports an interesting advance in the study of the supply of electrons to LH1-RC complexes with bound, four-heme cytochromes.

1. The title states that this co-structure reveals a mechanism for energetically "uphill" interprotein electron tunnelling, but it isn't clear what this mechanism is, and I wonder if 'mechanism' might be overstating things, particularly if the authors mean something involving Leu63. Perhaps 'route' or pathway' might be better. 'Mechanism' might imply a study with site directed mutants, mutant structures and extensive kinetics, for example.

Our response:

We appreciate the reviewer's positive assessment of our work. We have changed the title by adding the word "pathway" according to the reviewer's suggestion.

Reviewer #1's comments: Point 2

2. Page 4. Results, and also Methods. The co-crystallisation conditions don't specify the redox state of the HiPIP, but presumably it is in the reduced form.

Our response:

We have specified in the Results (p. 4) and Methods (p. 12) that the absorption spectrum (Supplementary Fig. 1) showed that the HiPIP was purified in the reduced state, according to the reviewer's suggestion.

Reviewer #1's comments: Point 3

3. Page 4. The call to Figure S3 on page 4 does not mention the comparison with the HiPIP in other species shown in Fig S3, but the existence of the alignments could be flagged up here.

Our response:

We have added a sentence to mention that the amino acid sequences (p. 4) of HiPIP were compared between *Tch. tepidum* and other species in Supplementary Fig. 3.

Reviewer #1's comments: Point 4

4. Page 7 - Predicted Electron Transfer Pathway. The authors propose that electron transfer from 4Fe-4S to heme-1 is mediated by the sidechain of Leu63 in HiPIP. The authors could comment on how well conserved this residue is and point the reader to supplementary Figure S3. There is nothing noteworthy about Leu63, and presumably any residue in this position would play the same role, by filling the intervening space; Leu63 does not physically accept and donate electrons. The Dutton-

Moser 'ruler' (ref 36) shows that the distance between the donor and acceptor is the main determinant of the rate of electron tunnelling, and as long as the distance requirement is satisfied, as it is here, the rate of ET will be adequate. As the authors point out, the measured ET rate for the Tch. tepidum HiPIP/RC pair is much slower than calculated because formation (and likely dissociation) of the ET complex is rate limiting. Thus, Leu63 is not functionally important, and certainly not physiologically so.

Our response:

We agree with the reviewer's comment on this point and have added sentences (p. 11) in the Discussion to mention that the bridging residue could be variable and the distance between the donor and acceptor is the main determinant of the rate of electron tunneling, as suggested by the reviewer.

Reviewer #1's comments: Point 5

5. The lines in figure S7 for the protein are very difficult to see. It may be worthwhile to use a white background and/or increase the line width in this figure.

Our response:

The images in Supplementary Fig. 7 were direct screen-shot outputs from the *Pathways* plugin software package and were intended to schematically show the alternative routes of electron transfer with different coupling strengths. The most likely pathways were extracted and illustrated in Fig. 5. We tried a change of the backgrounds to white, but when we did, there was no improvement. Instead, we have enlarged these images for easier inspection. As can be seen, the lines for the proteins in Supplementary Fig. 7 are now much more apparent.

Reviewer #1's comments: Point 6

6. Page 9. The authors describe ITC experiments in which HiPIP was titrated to a molar ratio of 0.8, much lower than the 15:1 used in the study. Did the authors see any interesting behaviour at higher molar ratios and could an affinity be determined from the data? If so how does the affinity compare to previous studies?

Our response:

The molar ratio of HiPIP:LH1-RC=15:1 was an experimentally optimized value to obtain the co-crystals that diffracted to the highest resolution. Interactions between the two proteins occur at much lower ratios as can be seen from the ITC profiles in Supplementary Fig. 8. However, it was difficult to quantitatively evaluate an affinity at lower concentrations of HiPIP because the ITC measurement is highly sensitive not only to binding events but also to any process that results in heat loss/gain, such as diffusion of the proteins during titration, mix of different buffers and ion exchange between the solvent and the complexes (Ref. 25). Thus, a reliable affinity value can only be achieved if an experimental system were available to eliminate or at least to minimize non-binding events at very low concentrations; at present, such a system does not exist.

Reviewer #1's comments: Point 7

7. The discussion is focused on the uphill energy transfer from HiPIP to Heme-1 and quotes potentials from previous studies. Have the authors considered whether bending of Heme-1 in the RC-HiPIP

complex may shift the reduction potential of Heme-1 and make the electron transfer from HiPIP more energetically favourable?

Our response:

Heme distortion has been suggested to modulate various properties of the heme group including the reduction potential. It has been demonstrated that decreasing heme distortion results in a decrease in its reduction potential (Ref. 39). This infers that bending of heme-1 in the RC-HiPIP complex should increase its reduction potential, making it energetically more favorable to accept electrons from HiPIP by reducing the uphill gap. However, it should be pointed out that effect of the heme distortion on reduction potential may be smaller compared to the effects of electrostatic interactions and/or hydrophobicity around the heme pocket (Ref. 40). There is a strong correlation between the reduction potential and the surrounding hydrophobicity: reduction potential increases with decreasing heme exposure to solvent (Ref. 41). Based on this result, the reduction potential of heme-1 in the RC-HiPIP complex is expected to increase because the solvent-exposed edge of the heme-1, as seen in Supplementary Figs. 5e and 5f, is covered by the hydrophobic surface of the HiPIP, leading to an increased hydrophobicity upon HiPIP binding. This effect again could reduce the uphill gap, making the heme-1 more favorable for accepting electrons from HiPIP. We thank the reviewer for pointing out this issue and have added a paragraph and three references (Ref. 39-41) in the Discussion section (p. 11) to address this issue.

Reviewer #1's comments: Minor corrections

Minor corrections:
Abstract: "Structure of the co-complex" to "The structure of the co-complex"
Abstract: "RC in close vicinity" to "RC in close proximity"
Page 3: "group of soluble proteins that shuttle between the RC and…" to "group of soluble proteins that shuttle electrons between the RC and…"
Page 4: "Our structural results here verify…" to "Our structural results verify…"
Page 4: "…and HiPIP but go…" to "…and HiPIP and go…"
Page 4 "…from Glu60 to Asp120 and in close vicinity…" to "…from Glu60 to Asp120 and in close proximity…"
Page 5 "…exposed to the solvents." to "Exposed to the solvent"
Page 9. Abbreviation ITC is not defined
Fig 1 legend. A in BChl a should be italicised
Fig 4 legend 'room-mean' to 'root-mean'

Our response:

We have corrected all of these wordings (indicated by the red fonts) as pointed out by the reviewer.

Reviewer #2

Reviewer #2's comments: Point 1

The topic of the article is relevant and is of interest to the researchers in the area of bacterial photosynthesis as well as to anyone interested in biological electron transport. The undoubted

achievement of this work is the successful crystallization of the short-lived complex HiPIP:LH1-RC and the resolving of its structure with relatively high resolution 2.9 Å. All main conclusions of the manuscript are based on this structure. My main questions, as a reviewer, are also related to the structure.
To start with, authors pay special attention to the B-factor, and it is used to color Figs. 1b and S4. However its value in the HiPIP:LH1-RC structure varies from 123 to 261 and seems to be extremely high for such a resolution. At the same time, too low values of R-factors are noted, which may be related to inaccurate B-factor refinement. The fact that the atoms inside of a protein have lower B-factor (since their positions are more stable) compared to the atoms on the surface of a protein, is known and, to my opinion, does not deserve special attention.

Our response:

- We appreciate the reviewer's positive assessment of our work. The B-factor is a frequently asked question from the reviewers for our previously published 3.0-A and 1.9-A LH1-RC structures. This is because this complex has a total number of 36 proteins: 32 of them are short chains containing 50~60 residues and their relatively soluble termini are extruded from the membrane surface with disordered conformations; two proteins (C- and H-subunits) have large membrane-extruded soluble domains. These compositional and topological features are unique among large membrane protein complexes with similar molecular weights (~400 kDa) and contributed to the relatively high overall values of the B-factor compared to those of the structures of protein complexes with similar sizes and analyzed at the same resolution. The small soluble HiPIP (~9kDa) is bound to the edge of the large soluble C-subunit, and the value of B-factor is also slightly dependent on the parameters used in the refinement process. For these reasons, we intended in our work to illustrate, using a gradient colour scheme, the <u>large difference</u> in the B-factors of the HiPIP between its binding surface and the opposite solvent-exposing side rather than stating absolute values. Therefore, we only briefly mentioned the uneven B-factor distribution for HiPIP and avoided citing their absolute values in the text (p. 5).
- The HiPIP:LH1-RC structure was refined by the *Phenix.refine* software package using standard TLS parameters. The B-factors obtained were in ranges of 59.2–261.2 (Mean:128.7 in Table 1) for all atoms, 60.0–261.2 (Mean:128.1 in Table 1) for macromolecules, and 59.2–220.3 (Mean:131.6 in Table 1) for ligands. These values are slightly dependent on the parameters used in the refinement, and the lower limit values (~60 A^2) are in reasonable range for a typical integral membrane protein complex with similar molecular weights and analyzed at the same resolution. The atoms with relatively low B-factors are mainly located in the rigid core of the reaction center, and they have highly stable conformations as pointed out by the reviewer.

Reviewer #2's comments: Point 2

Further, when specifying distances between atoms in the structure, coordinate error for this resolution are usually shown. In your manuscript, they are mentioned neither in the text, nor in the figures and tables. I can offer a reference on the topic - A.A.Vaguine, J. Richelle, S.J.Wodak, SFCHECK: a unified set of procedures for evaluating the quality of macromolecular structure-factor data and their agreement with the atomic model, Acta Crystallogr. D: Biol. Crystallogr. 55 (1999) 191–205. One can expect that the coordinate error at resolution 2.9 Å may be quite large, perhaps at least 0.27-0.29 Å.

Our response:

We agree with the reviewer's comment and have added a description on the estimated coordinate error in the revised text (p. 5) with a reference (Ref. 14).

Reviewer #2’s comments: Point 3

I believe that it is hardly correct to make superposition using structures with so different resolutions, 2.9 Å for HiPIP:LH1-RC complex and 1.5 Å for HiPIP protein or 1.9 Å for LH1-RC complex (Figs 4 and S6). It can be noticed that the deviations found in the positions of prosthetic groups or protein are pretty close to the value of the expected coordinate error of the HiPIP:LH1-RC structure.
Thus, most of the conclusions about hydrogen-bonds, distances between amino acid residues, deviations of prosthetic groups and the protein of the cytochrome subunit do not seem very convincing and need to be clarified.

Our response:

We attempted to emphasize the structural differences of the deviation from reference structures between different portions within the same protein induced by HiPIP binding. Despite different resolutions between our co-complex and corresponding individual proteins, it is clear that the structural changes occurred in an increasing manner toward the binding domain and reached maximum on the interacting surface between the C-subunit and HiPIP (Fig. 4). These changes including for the prosthetic groups are considered to be obviously large even by taking into account the coordinate errors. As we have added coordinate error in the revised text, the errors in hydrogen bonds, distances between residues, deviations of prosthetic groups and the protein of the Cyt-subunit could be estimated from this information. In this work, we only picked up relatively strong hydrogen bonds, large distances between residues and large deviations for the Cyt-subunit.

Reviewer #2’s comments: Point 4

I also have small remarks. Figure 1 is hard to read with too many colors. Similar colors were selected to represent different objects (for example, purple and magenta, Fe-S - yellow-orange ball; Ca - orange ball). Table S1 is shown twice, on the pages 23 and 37.

Our response:

- The HiPIP:LH1-RC co-complex contains 7 different types of proteins and 8 different types of prosthetic groups. Figure 1a was only intended to show schematically the overall structure and we tried to use minimum number of simple colors available to get maximum contrast for different parts, while Fig. 1b is the main figure to show up the interface between the two interacting proteins. The color for Fe-S is yellow-orange, but the color for Ca^{2+} was actually gold. We have changed the description for the Ca^{2+} in Fig. 1 caption, and thank the reviewer for pointing out this issue.
- We have corrected this error on Table S1, now the Supplementary Table 1 in the revised manuscript.

Reviewer #3

Reviewer #3’s comments: Point 1

In this study a crystal structure at a resolution of 2.9 Å was determined of a complex (contact surface 617 Å2) between the high potential iron-sulfur protein (HiPiP) and the reaction center (RC) isolated from the thermophilic purple sulfur bacterium Tch. Tepidum. Optimum/successful crystallization conditions required an excess of HiPiP, and an HiPiP: RC ratio of approximately 15 at pH 5.

This is an interesting study, but the text has a number of incorrect/questionable statements. Chronologically:
1. p7, l6. Vague-poor form for referencing: "…dominated by the 4Fe-4S cluster (see later)." What does "later" mean?

<u>Our response:</u>

We appreciate the reviewer's interest in our work, and have removed the words "(see later)". The details were described in the following section (**Predicted electron transfer pathway**, p.7 from last sentence: Based on its…) and also in the Discussion section (p. 11, last paragraph: Based on the…).

<u>Reviewer #3's comments: Point 2</u>

2. Errata: (1) Results, l3: Fig. S2 not S1, S2.; (2) p7, l6. "…superimposing the…?"; (3); p7, l9: "…binding of ferredoxin."

<u>Our response:</u>

We have corrected these typos.

<u>Reviewer #3's comments: Point 3</u>

3. p10. What is the reference for the statement about a '0.7 eV reorganization energy? A formula for the rate of electron transfer and its parameter dependence is not provided.

<u>Our response:</u>

We have added the reference (Ref. 37) for the reorganization energy. The empirical formula was also given in this reference.

<u>Reviewer #3's comments: Point 4</u>

4. In the manuscript, why are manuscript references 1-37 separated from refs 38-52.

<u>Our response:</u>

This was due to an editorial reason. We have combined the reference lists into one in the revised manuscript.

<u>Reviewer #3's comments: Point 5</u>

5. Fig. 4C. The colors should be changed so that the contrast between the 2 conformations of the heme can be seen.

<u>Our response:</u>

In order to show relative orientation (conformation) of the highly symmetric structure of the heme, we need to show the positions of the oxygen atoms (that are conventionally in red color) in the carboxyl groups. The colors (deepteal and grey) used to distinguish the two heme

groups including the oxygen atoms are considered to be the best for giving the highest contrast after consultation with our co-authors.

<u>Reviewer #3's comments: Point 6</u>

6. Fig. 5a is perhaps the most important figure in the manuscript as it relates to the major heading of the manuscript, an extended electron transfer pathway However, one cannot appreciate it because there is no information provided on the distances between the redox centers nor on the redox potentials of the Centers.
With this limited information and none in particular on the redox potentials of the carriers in Fig. 5a, nor on the distances between them it is not clear how this pathway can be characterized as "energetically 'uphill.'"

<u>Our response:</u>

As we described in the text, the reduction potentials for the *Tch. tepidum* HiPIP and heme-1 were determined to be +340 mV and +150 mV, respectively. The distances between the redox centers were given in Supplementary Table S2. The difference in the reduction potential means that the electron transfer from HiPIP to heme-1 is energetically "uphill". Actually, the software *Pathways* plugin does not require information of the reduction potential for prediction of the pathways. Therefore, Fig. 5a was generated only using the structural details. The reduction potentials combined with structural geometry were used for estimating electron transfer rates based on the well-known empirical formula as described in the Discussion section. To further make these clear, we have added a sentence on the distances between redox centers in the text (p. 7) and added reduction potentials for the HiPIP and the heme-1 in Fig. 5b.

<u>Reviewer #3's comments: Point 7</u>

7. Similarly, it is not clear that there is enough information in Fig. S7 to allow appreciation of nuances of electron transfer in this study. Thus, in its present form, the manuscript is not acceptable for publication.

<u>Our response:</u>

The images in Supplementary Fig. 7 were direct screen-shot outputs from the *Pathways* plugin software package and were intended to schematically show the alternative routes of electron transfer with different strengths. The most likely pathways were extracted and illustrated in Fig. 5. We have made these images larger for an easier inspection. We have also added the coupling strengths in Supplementary Table 2 for the alternative routes shown in Supplementary Fig. 7.

<u>Reviewer #3's comments: Point 8</u>

8. The manuscript needs a Table of Abbreviations.

<u>Our response:</u>

This journal does not request a table of abbreviations, but we give it here and can add it to the ms if the reviewer and editor request it:

Alc: Allochromatium, BChl: bacteriochlorophylls, Cyt: cytochrome, HiPIP: high-potential iron-sulfur protein, LDAO: lauryl dimethylamine N-oxide, LH1: core light-harvesting complex, MALDI/TOF-MS: matrix-assisted laser desorption/ionization time-of-flight mass spectroscopy, OPC: *n*-octyl-phosphocholine, PEG: polyethylene glycol, RC: reaction center, *Rb: Rhodobacter, Tch: Thermochromatium*.

REVIEWER COMMENTS

Reviewer #1 (Remarks to the Author):

The authors have answered the points raised, and they have attended to the suggested alterations and corrections.

Neil Hunter and David Swainsbury

Reviewer #2 (Remarks to the Author):

1) As I said in my previous review, the main achievement and basis of this work is successful crystallization of the short-lived complex HiPIP:LH1-RC. Provided that it was done correctly, which I doubt. As noted by other referees, crystals were grown at very high molar ratio of HiPIP to LH1-RC (15:1) and also at pH 5.0, which is not physiological pH. I suspect that under such conditions there is a high probability of non-specific binding. The authors themselves admit that the interaction between the HiPIP and LH1-RC proteins is weak, and the affinity depends on many factors (ref. 25). Since the hydrophobic binding pocket on the THC subunit is flat, even a slight shift let alone a considerable rotation of HiPIP on this surface could change the real picture of protein interactions. Authors cite three papers that presented models of the complex between THC and HiPIP for few purple bacteria (ref. 21,22,5). Some models were calculated using protocols of homology building and protein docking (ref. 5, 21). The one proposed for Ru. gelatinosus derived from using manual docking simulations (ref. 22). Even though the interaction site was the same, the relative orientation of the two proteins in models calculated by two different methods was dissimilar. It has been suggested that the described models for the HiPIP–THC complexes may not represent the only specific structure for which the redox reaction occurs, and multiple possible associations with different electron transfer capabilities could in fact be operative in vivo (ref. 5). To my surprise, the HiPIP - LH1-RC structure described in this manuscript is highly consistent with the model calculated for Tch. tepidum (ref. 5) in many details concerning the interaction surface of complexes as well as the electron transfer pathways calculated basing on this model.
2) Even if I had no doubts about the specificity of protein binding in the short-lived complex, I disagree with the conclusions the authors draw based on the presented structure.
page 5 – "Coordinate error was estimated to be 0.50 Å based on the maximum-likelihood refinement (ref. 14)"
This is a very high coordination error for such a resolution. Usually for a given resolution it does not exceed 0.3 Å. This forces us to take another close look at the refinement statistics data (Table 1). Although CC1/2 is 0.334 for the upper range, all other parameters like I / σ = 0.69 and R-merge = 2.06 seem to be absolutely invalid. The data indicate that the real resolution of this set is significantly lower, not better than 3.5 Å.
I believe that with such a low resolution and with such a coordination error, it is completely incorrect to draw a picture of interactions on the contact surface, to talk about formation of "strong" and "weak" H-bonds, to estimate distances between amino acids (Table 1, Supplementary) and even more so to use this structure to build the path of the electron transfer. The authors used a convenient but incorrect way to get around the problem of low resolution: in the HiPIP:LH1-RC low resolution structure they replaced HiPIP-part with the high resolution HiPIP structure (0.48 Å). The same goes for the heme "bending" (Figure 4c), as well as for the structural changes of the 4Fe-4S cluster. If a map of electron density for heme-1 were shown, it would be possible to see how justified this bend is. High resolution structures can be used only as search models, but comparison of structures with such different resolutions, and even more so such a substitution as made in this work, can produce misleading results and incorrect conclusions. I already mentioned this in my first review, but did not receive any comments.
Thus, in spite of the fact that the results of the work are consistent with the previously proposed model (ref. 5), I believe that these results are not convincing enough to be published in Nature Communications.

Response to reviewer #2:

Reviewer #2's comments: Point 1

1) As I said in my previous review, the main achievement and basis of this work is successful crystallization of the short-lived complex HiPIP:LH1-RC. Provided that it was done correctly, which I doubt. As noted by other referees, crystals were grown at very high molar ratio of HiPIP to LH1-RC (15:1) and also at pH 5.0, which is not physiological pH. I suspect that under such conditions there is a high probability of non-specific binding. The authors themselves admit that the interaction between the HiPIP and LH1-RC proteins is weak, and the affinity depends on many factors (ref. 25). Since the hydrophobic binding pocket on the THC subunit is flat, even a slight shift let alone a considerable rotation of HiPIP on this surface could change the real picture of protein interactions. Authors cite three papers that presented models of the complex between THC and HiPIP for few purple bacteria (ref. 21,22,5). Some models were calculated using protocols of homology building and protein docking (ref. 5, 21). The one proposed for Ru. gelatinosus derived from using manual docking simulations (ref. 22). Even though the interaction site was the same, the relative orientation of the two proteins in models calculated by two different methods was dissimilar. It has been suggested that the described models for the HiPIP–THC complexes may not represent the only specific structure for which the redox reaction occurs, and multiple possible associations with different electron transfer capabilities could in fact be operative in vivo (ref. 5). To my surprise, the HiPIP - LH1-RC structure described in this manuscript is highly consistent with the model calculated for Tch. tepidum (ref. 5) in many details concerning the interaction surface of complexes as well as the electron transfer pathways calculated basing on this model.

Our response:

- Strictly, almost all X-ray crystallographic structures are obtained using the crystals formed under non-physiological conditions, and the diffraction data are collected at extremely low temperatures (typically ~100K). Especially for the integral membrane protein complexes, non-natural detergents are usually used for solubilization and crystallization in addition to precipitant agents (also non-natural) and salts. The HiPIP and C-subunit in the RC are located on the periplasmic side of the photosynthetic membrane where proton concentration is higher than that on the cytoplasmic side due to formation of a proton gradient under light-illuminating condition. The pH difference between the two sides of the chromatophore membranes from *Rb. capsulatus* was estimated to be up to ~ 3 full units under such conditions (see the reference listed below, Baccarini-Melandri et al. 1977). Considering that the pHs on the cytoplasmic side of the membrane are typically in the range of 7–8, the pH5 used in our co-crystallization of the HiPIP:LH1-RC should be very close to, if not the same as, physiological conditions.
- We did conduct a control experiment using the LH1-RC-alone crystals formed at pH5 under the same crystallization condition as that for the co-complex. The structure of the LH1-RC at pH5 was solved with a *C*2 space group and refined to 2.5 Å resolution. The RMSD was 0.424 Å for the secondary structures between the LH1-RC(pH5) alone and the LH1-RC in the HiPIP:LH1-RC co-complex. Similarly, the RMSD was 0.421 Å between LH1-RC in the HiPIP:LH1-RC co-complex and the published LH1-RC structure (pH6.2, 1.9-Å resolution, PDB ID: 5Y5S). The results indicate that both pH and excess of HiPIP in the ranges of our study do not affect overall structure of the LH1-RC significantly in the co-complex. The only detectable deviation was observed on the binding surface of the Cyt-subunit as described in the manuscript (Fig. 4, p. 6-7 and 11), which was caused by the binding of HiPIP.
- It is well accepted that crystallographic structures represent most probable conformation of the protein complexes and therefore an extracted "real picture" of conformational

ensemble. Although interactions between the LH1-RC and HiPIP are weak, the co-crystal structure still represents the *most probable* snapshot (natural picture) over a structural ensemble because we can trace the main chains at the initial stage of model building and finally determine the side-chain conformation for most residues in the HiPIP from the electron density maps (Fig. 2c). This cannot be achieved if the HiPIP was bound in a highly non-specific and/or disordered manner. We do not exclude the possibility of alternative binding modes but if they exist, they should occur in much lower probabilities compared to the structure determined in our study. We consider the capture of a co-complex formed mainly by weak hydrophobic interactions as one of the highlights of this work because such a co-complex structure has not been previously reported.

- We cannot comment on the statement of the reviewer that "To my surprise, the HiPIP-LH1-RC structure described in this manuscript is highly consistent with the model calculated for Tch. tepidum (ref. 5) …" because coordinates of the calculated co-complex structure are not available in ref. 5. Without the coordinates, we cannot make a quantitative comparison (such as RMSD) and judge whether or not our structure is "highly consistent" with the calculated one. However, as mentioned in the Discussion (p. 11-12), the simulation (ref. 21) for the HiPIP:Cyt-subunit of *Alc. vinosum* (a close relative of *Tch. tepidum* with high sequence identities of 87% for the HiPIP and 78% for the Cyt-subunit, Supplementary Fig. 3) predicted a much different binding structure from our co-crystal structure. For example, the conserved aromatic ring of Phe48 (a key residue on the interface) in *Alc. vinosum* HiPIP was almost parallel to the plane of the heme-1 group (ref. 21); this is in contrast to that in the *Tch. tepidum* HiPIP:LH1-RC structure where the two planes are inclined by 55° toward each other (Fig. 2c). Moreover, the contact surface area of the *Tch. tepidum* HiPIP:LH1-RC in our study was found to be significantly smaller than that calculated for the HiPIP-docked RC of *Alc. vinosum* (ref. 21). This points to difficulties in docking-model-based calculations that do not fully account for weak hydrophobic interactions. All calculated docking structures can only be viewed as rough models and need to be verified by the experimentally determined structures in cases for discussing structural details.

Reviewer #2's comments: Point 2

2) Even if I had no doubts about the specificity of protein binding in the short-lived complex, I disagree with the conclusions the authors draw based on the presented structure.
page 5 – "Coordinate error was estimated to be 0.50 Å based on the maximum-likelihood refinement (ref. 14)"
This is a very high coordination error for such a resolution. Usually for a given resolution it does not exceed 0.3 Å. This forces us to take another close look at the refinement statistics data (Table 1). Although CC1/2 is 0.334 for the upper range, all other parameters like I / σ = 0.69 and R-merge = 2.06 seem to be absolutely invalid. The data indicate that the real resolution of this set is significantly lower, not better than 3.5 Å.
I believe that with such a low resolution and with such a coordination error, it is completely incorrect to draw a picture of interactions on the contact surface, to talk about formation of "strong" and "weak" H-bonds, to estimate distances between amino acids (Table 1, Supplementary) and even more so to use this structure to build the path of the electron transfer. The authors used a convenient but incorrect way to get around the problem of low resolution: in the HiPIP:LH1-RC low resolution structure they replaced HiPIP-part with the high resolution HiPIP structure (0.48 Å). The same goes for the heme "bending" (Figure 4c), as well as for the structural changes of the 4Fe-4S cluster. If a map of electron density for heme-1 were shown, it would be possible to see how justified this bend is. High resolution structures can be used only as search models, but comparison of structures with such different resolutions, and even more so such a substitution as made in this work,
can produce misleading results and incorrect conclusions. I already mentioned this in my first review, but did not receive any comments.

Thus, in spite of the fact that the results of the work are consistent with the previously proposed model (ref. 5), I believe that these results are not convincing enough to be published in Nature Communications.

Our response:

- Resolution cutoff is a long-standing controversial, multi-factor issue in macromolecular X-ray crystallography and there is no single "correct" cutoff for every case (see the following paragraph). Both the resolution limit and coordinate error are global factors that are related to each other. These values, along with the *B*-factors, also depend on the refinement software packages and the parameters used. We have tested *Phenix.refine* and *Refmac5* in CCP4 for our structure at 2.9 Å using the parameter sets as similar as possible. We found a difference of about 0.1 Å at this resolution for the overall coordinate errors (maximum likelihood) between the two programs. The coordinate error actually depends on the resolution (resolution cutoff) of a refined structure. For example, the structure of *Tch. tepidum* LH1-RC was first determined at 3.0 Å (PDB ID: 3WMM) (Niwa et al. 2014) for which the coordinate error was 0.62 Å. This value decreased to 0.24 Å when the resolution was later improved to 1.9 Å (ref. 13, PDB ID: 5Y5S). We suppose that the 0.3 Å-limit of the coordinate error mentioned by the reviewer may be more or less for "relatively high" resolution structures toward the "end" of refinement.
- Following the milestone paper published in *Science* (Karplus and Diederichs 2012), a series of studies have been addressing the resolution cutoff issue and challenging its traditional criterion by pointing out that (i) R-merge and I/σ values are poorly suited for determining the high-resolution limit, (ii) the so-called "standard protocols" discard much useful data, and (iii) CC*(CC1/2) is a single statistically valid guide for deciding which data are useful (Diederichs and Karplus 2013; Wang and Wing 2014; Lou et al. 2014; Karplus and Diederichs 2015). Our CC1/2 value is within the recommended range, as Reviewer #2 has acknowledged. It has been demonstrated that R-merge should *not* be used for guiding cutoff decisions and weak reflections with I/σ values as low as 0.3 may still contain useful information (Karplus and Diederichs 2015; Hegde et al. 2017).
- In addition to the above metrics, we adopted an electron density map as a critical assessment of quality control of our data. The figures below compare the density maps (2mFo–DFc: 0.92σ; mFo–DFc: 3.02σ) using the *Tch. tepidum* LH1-RC structure (PDB ID: 5Y5S) as search model (represented by sticks for a portion of the Cyt-subunit) and refined to resolutions at 2.9 Å (right, this work) and 3.5 Å (left) as estimated by the reviewer.

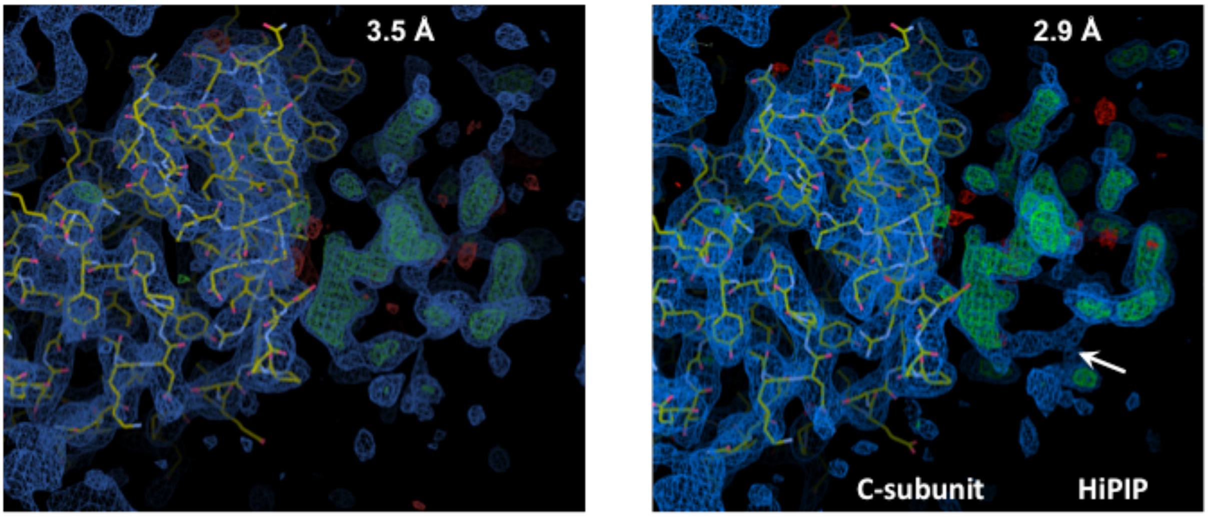

The 2.9-Å data set clearly yielded higher quality map connectivities for the HiPIP portion (the arrow indicates an example) than that of 3.5-Å data. This advantage is particularly crucial at

the initial stage of model building when trying to trace main-chain conformation of the bound HiPIP because automatic molecular replacement cannot yield a correct conformation for the HiPIP at this stage. The 3.5-Å data set excluded about 40% reflections from the 2.9-Å data set and resulted in a 4% decrease in the real-space overall CC value. This indicates that the 2.9-Å data indeed contained useful information despite many weak reflections. Some of this effect on the map quality can be viewed from the above figures and the figures presented in the next paragraph. We have added some details on the initial model building of the bound HiPIP structure in the Methods section (p. 13)

- As requested by Reviewer #2, we provide electron density maps (2mFo–DFc: 0.92σ) here for the heme-1 group and surrounding residues at 3.5-Å (left) and 2.9-Å (right) resolutions. The 2.9-Å map is also added to our manuscript as Supplementary Fig. 6(a).

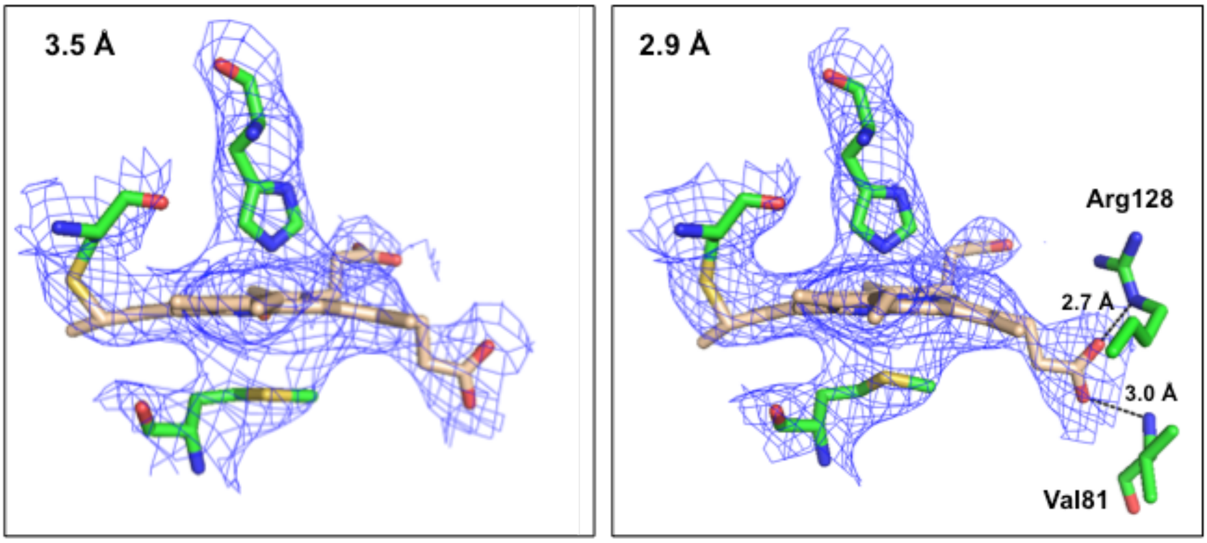

One of the carboxyl groups in heme-1 forms hydrogen bonds with C-Arg128 and C-Val81, which may contribute to the heme-bending.

- The comment of the reviewer "…they replaced HiPIP-part with the high resolution HiPIP structure" is a misunderstanding. The HiPIP structure (PDB ID: 1EYT) used in our structure determination was at 1.5-Å resolution (p. 13 and figure caption in Fig. 4). The ultra-high-resolution structure (0.48 Å) was only mentioned in the Discussion section. We have added "1.5-Å resolution" to indicate this in the Methods section (p. 13).

References for this response

Baccarini-Melandri A, Casadio R, Melandri BA (1977) Thermodynamics and kinetics of photophosphorylation in bacterial chromatophores and their relation with the transmembrane electrochemical potential difference of protons. Eur J Biochem 78:389-402

Diederichs K, Karplus PA (2013) Better models by discarding data? Acta Cryst D69:1215-1222

Hegde RP, Fedorov AA, Sauder JM, Burley SK, Almo SC, Ramagopal UA (2017) The hidden treasure in your data: phasing with unexpected weak anomalous scatters from routing data sets. Acta Cryst F73:184-195

Karplus PA, Diederichs K (2012) Linking crystallographic model and data quality. Science 336:1030-1033

Karplus PA, Diederichs K (2015) Assessing and maximizing data quality in macromolecular crystallography. Curr Opin Struct Biol 34:60-68

Lou Z, Rajashankar K, Dauter Z (2014) Weak data do not make a free lunch, only a cheap meal. Acta Cryst D70:253-260

Niwa S, Yu L-J, Takeda K, Hirano Y, Kawakami T, Wang-Otomo Z-Y, Miki K (2014) Structure of the LH1-RC complex from *Thermochromatium tepidum* at 3.0 Å. Nature 508:228-232
Wang J, Wing RA (2014) Diamonds in the rough: a strong case for the inclusion of weak-intensity X-ray diffraction data. Acta Cryst D70:1491-1497
